# Use of Prokinetic Agents in Adult ICU Patients: An International Inception Cohort Study (PATIENCE)

**DOI:** 10.1111/aas.70290

**Published:** 2026-06-24

**Authors:** Vera Crone, Morten Hylander Møller, Anders Granholm, Anders Perner, Waleed Alhazzani, Laura Rindom Krogsgaard, Abdulrahman Al‐Fares, Johanna Hästbacka, Marlies Ostermann, Carmen A. Pfortmueller, Ricard Ferrer, Annika Reintam Blaser, Olof Wall, Eric Keus, Wojciech Szczeklik, Peter Martin Hansen, Jens Michelsen, Mikkel Bak, Lise Skyttegaard Balkert, Tino Severinsen, Anne Craveiro Brøchner, Jonas Elmer Pedersen, Anne Sofie Andreasen, Camilla Tageby Nielsen, Theis Skovsgaard Itenov, Maj‐Brit Nørregaard Kjær, Zemira Engbakken, Morten Heiberg Bestle, Peter Buhl Hjortrup, Christian Juhl Svendsen, Sandra Lindkvist‐Viggers, Björn Anders Brand, Andreas Bender Jonsson, Tobias Browall Krogh, Kristian Reinhold Jauho, Mathias Sinkbæk Thomsen, Therese Simonsen Straarup, Eva Poulsen, Christian Gade Nissen, Olena Breum, Per Lund Petersen, Christopher Torp, Bodil Steen Rasmussen, Anne‐Marie Gellert Bunzel, Seraina‐Flavia Buholzer, Elizabeth Hardman, Daniel Törnberg, David S. Pérez, Kenneth van Smaalen, Marisa Onrust, Liivi Maddison, Oskar Appelberg, Hans‐Erik Ehrlich, Kadri Tamme, Reile Juhanson, Jaanus Korjas, Ville Jalkanen, Marleena Finni, Matti Reinikainen, Talvikki Koskue, Marcelina Czok, Pawel Zatorski, Oliwia Doroba, Bartosz Kudlinski, Pawel Nowak, Szymon Bialka, Anna Kluzik, Hanna Baran, Zainab Al Duhailib, Hakeam A. Hakeam, Mohammed Alshahrani, Osama Ahmed Elfaki, Fadah A. Alanazi, Rakan Aldhahri, Khairallah Belkhouja, Ali Alahmari, Rawabi M. Alsayer, Ola Friman, Anca Balintescu, Mette Krag

**Affiliations:** ^1^ Department of Intensive Care Holbæk Hospital Holbæk Denmark; ^2^ Department of Intensive Care Copenhagen University Hospital—Rigshospitalet Copenhagen Denmark; ^3^ Department of Clinical Medicine University of Copenhagen Copenhagen Denmark; ^4^ Section of Biostatistics, Department of Public Health University of Copenhagen Copenhagen Denmark; ^5^ Research and Innovation Institute Ministry of Defense Health Services Riyadh Saudi Arabia; ^6^ Critical Care and Internal Medicine Department, College of Medicine Imam Abdulrahman Bin Faisal University Dammam Saudi Arabia; ^7^ Gastrounit, Section of Gastroenterology Hvidovre Hospital Hvidovre Denmark; ^8^ Department of Anaesthesia, Critical Care Medicine and Pain Medicine Al‐Amiri Hospital, Ministry of Health Kuwait City Kuwait; ^9^ Department of Intensive Care Tampere University Hospital, Wellbeing Services County of Pirkanmaa and Tampere University Tampere Finland; ^10^ Department of Critical Care King's College London, Guy's and St. Thomas' NHS Foundation Trust London UK; ^11^ Department of Intensive Care Inselspital, Bern University Hospital and University of Bern Bern Switzerland; ^12^ Department of Intensive Care Vall d'Hebron Hospital Universitari, Vall d'Hebron Barcelona Hospital Campus Barcelona Spain; ^13^ Department of Anaesthesiology and Intensive Care University of Tartu Tartu Estonia; ^14^ Department of Intensive Care Medicine Lucerne Cantonal Hospital Lucerne Switzerland; ^15^ Department of Anaesthesia and Intensive Care Danderyds Sjukhus Stockholm Sweden; ^16^ Department of Critical Care University Medical Center Groningen Groningen the Netherlands; ^17^ Center for Intensive Care and Perioperative Medicine Jagiellonian University Medical College Krakow Poland; ^18^ Department of Intensive Care Odense University Hospital Svendborg Denmark; ^19^ Department of Intensive Care Odense University Hospital Odense Denmark; ^20^ Department of Intensive Care University Hospital of Southern Denmark Aabenraa Denmark; ^21^ Department of Anaesthesiology and Intensive Care University Hospital of Southern Denmark Kolding Denmark; ^22^ Department of Regional Health Research, Faculty of Health Scienc es University of Southern Denmark Odense Denmark; ^23^ Department of Anaesthesiology and Intensive Care Lillebælt Hospital Vejle Denmark; ^24^ Department of Anaesthesiology and Intensive Care Copenhagen University Hospital—Herlev‐Gentofte Hospital Herlev Denmark; ^25^ Department of Anaesthesiology and Intensive Care Copenhagen University Hospital—Bispebjerg and Frederiksberg Hospital Copenhagen Denmark; ^26^ Department of Anaesthesiology and Intensive Care Copenhagen University Hospital—North Zealand Hillerød Denmark; ^27^ Department of Cardiothoracic Anaesthesia and Intensive Care Copenhagen University Hospital—Rigshospitalet Copenhagen Denmark; ^28^ Department of Anaesthesiology and Intensive Care Copenhagen University Hospital—Amager and Hvidovre Hvidovre Denmark; ^29^ Department of Neuroanaesthesiology Copenhagen University Hospital—Rigshospitalet Copenhagen Denmark; ^30^ Department of Anaesthesiology and Intensive Care Copenhagen University Hospital—Herlev‐Gentofte Hospital Gentofte Denmark; ^31^ Department of Anaesthesiology and Intensive Care Zealand University Hospital Roskilde Denmark; ^32^ Department of Anaesthesiology and Intensive Care Zealand University Hospital Køge Denmark; ^33^ Department of Anaesthesiology and Intensive Care Zealand University Hospital Nykøbing Falster Denmark; ^34^ Department of Intensive Care Randers Regional Hospital Randers Denmark; ^35^ Department of Anaesthesiology and Intensive Care Viborg Regional Hospital Viborg Denmark; ^36^ Department of Intensive Care Aarhus University Hospital Aarhus Denmark; ^37^ Department of Intensive Care North Denmark Regional Hospital—Hjørring Hjørring Denmark; ^38^ Department of Intensive Care Gødstrup Hospital Gødstrup Denmark; ^39^ Department of Anaesthesiology and Intensive Care Aalborg University Hospital, Aalborg University Aalborg Denmark; ^40^ Department of Clinical Medicine Aalborg University Aalborg Denmark; ^41^ Department of Clinical Sciences Danderyd Hospital Stockholm Sweden; ^42^ Intensive Care Centre North Estonian Medical Centre Tallinn Estonia; ^43^ Department of Anaesthesiology and Intensive Care Tartu University Hospital Tartu Estonia; ^44^ Centre of Anaesthesiology and Intensive Care East Tallinn Central Hospital Tallinn Estonia; ^45^ Intensive Care Units Helsinki University Hospital Helsinki Finland; ^46^ Department of Anaesthesiology and Intensive Care Kuopio University Hospital, and University of Eastern Finland Kuopio Finland; ^47^ Intensive Care Unit Päijät‐Häme Central Hospital Lahti Finland; ^48^ First Department of Anesthesiology and Intensive Care Medical University of Warsaw Warsaw Poland; ^49^ Second Department of Anesthesiology and Intensive Care Poland Medical University of Warsaw Warsaw Poland; ^50^ Clinical Department of Anesthesiology and Intensive Care University of Zielona Góra Collegium Medicum Zielona Góra Poland; ^51^ Department of Anesthesiology and Intensive Care Medical University of Silesia in Katowice Katowice Poland; ^52^ Clinical Department of Anaesthesiology, Intensive Care and Pain Management Poznan University of Medical Science Poznań Poland; ^53^ Clinic of Anesthesiology and Intensive Therapy University Clinical Hospital No. 1 Named After Tadeusz Sokołowski in Szczecin Szczecin Poland; ^54^ Critical Care Medicine Department King Faisal Specialist Hospital and Research Centre Riyadh Saudi Arabia; ^55^ College of Medicine Alfaisal University Riyadh Saudi Arabia; ^56^ Pharmaceutical Care Division King Faisal Specialist Hospital and Research Centre Riyadh Saudi Arabia; ^57^ Critical Care Department King Fahad University Hospital, Imam Abdulrahman Bin Faisal University Dammam Saudi Arabia; ^58^ Department of Intensive Care Prince Sultan Military Medical City Riyadh Saudi Arabia; ^59^ Department of Intensive Care King Fahad Military Medical Complex Dhahran Saudi Arabia; ^60^ Department of Intensive Care King Fahad Armed Forces Hospital Jeddah Saudi Arabia; ^61^ Department of Intensive Care Armed Forces Hospital, Southern Region Khamis Mushayt Saudi Arabia; ^62^ Department of Population Public and Environmental Health, Ministry of Defense Health Services Riyadh Saudi Arabia; ^63^ Department of Intensive Care Karolinska University Hospital Stockholm Sweden; ^64^ Department of Clinical Science and Education, Section of Anaesthesia and Intensive Care, South General Hospital Karolinska Institute Stockholm Sweden; ^65^ Department of Anaesthesiology, Surgery and Trauma Centre Copenhagen University Hospital—Rigshospitalet Copenhagen Denmark

**Keywords:** erythromycin, feeding intolerance, intensive care, metoclopramide, prokinetic agents

## Abstract

**Background:**

Feeding intolerance is common in intensive care unit (ICU) patients, but evidence supporting prokinetic use is limited. We aimed to provide international epidemiological data on the use of prokinetic agents in adult ICU patients and to explore potential associations with patient‐important outcomes.

**Methods:**

We conducted an inception cohort study between August 2024 and March 2025 in acutely admitted ICU patients in 56 ICUs across 11 countries. The primary outcome was the proportion of patients receiving prokinetic agents. Secondary outcomes included associations with baseline characteristics, serious adverse events (SAEs), days alive out of ICU/hospital, days alive without life support and 90‐day mortality. Associations with SAEs, baseline characteristics and mortality were assessed using pre‐specified Cox regression models, while other secondary outcomes were evaluated using adjusted linear regression. All models were adjusted for country, severity of illness, number of comorbidities, surgery and ICU admission type.

**Results:**

Among 1440 ICU patients (median age 64 years, 56.9% male), 187 (13.0%; 95% confidence interval [CI] 11.3–14.8) received prokinetic agents during ICU stay, most commonly metoclopramide (65%). Prior abdominal surgery was associated with the initiation of prokinetic agents (hazard ratio [HR] 1.81; 95% CI 1.17–2.79). Use of prokinetic agents was statistically significantly associated with a higher hazard of experiencing a SAE (HR 1.9; 95% CI 1.3–2.8), fewer days alive out of ICU (mean difference [MD] −7.6 days; 95% CI, −13.4 to −2.2) and hospital (−13.5 days; 95% CI −18.7 to −8.4), but not with 90‐day mortality (HR 0.62; 95% CI 0.3–1.2).

**Conclusions:**

Prokinetic agents were used in 13% of ICU patients, most commonly metoclopramide and more often in those with prior abdominal surgery. Prokinetic use was associated with a higher hazard of experiencing SAEs and fewer days alive out of hospital/ICU.

## 1 Introduction

Enteral feeding intolerance is a frequent complication among patients in the intensive care unit (ICU), with reported prevalences ranging from 2% to 75% [1, 2]. Prokinetic agents may enhance upper gastrointestinal motility and facilitate nutritional uptake. However, supporting evidence for their effect in the ICU setting is limited [3].

Different prokinetic agents are used in the ICU [4]. Metoclopramide and domperidone are dopamine receptor antagonists that promote gastric emptying and enhance duodenal peristalsis. Erythromycin stimulates motilin receptors in the duodenum, inducing antral and duodenal contractions [3, 5], while prucalopride, a serotonin receptor agonist, promotes gastrointestinal transit [6].

A systematic review and meta‐analysis from 2016 of 13 randomised trials (1341 ICU patients) assessed the effect of prokinetic agents versus placebo or no intervention [3]. Prokinetic agents reduced feeding intolerance, but no statistically significant effect was observed on patient‐important outcomes, including mortality, pneumonia, ICU length of stay, diarrhoea and vomiting. No other adverse events were reported [3]. Other studies have associated prokinetic agents with cardiac arrhythmias and extrapyramidal symptoms, but data on the incidence of these events in ICU patients are scarce [6, 7].

Although prokinetic agents are recommended as part of the treatment for feeding intolerance, these recommendations are based primarily on a few heterogeneous trials with overall low certainty of evidence [8], and epidemiological data on the use of prokinetic agents in ICU patients are sparse.

With this international inception cohort study, we aimed to provide epidemiological data on the use of prokinetic agents in adult ICU patients and to explore potential associations with patient‐important outcomes.

We hypothesised that one in four acutely admitted ICU patients would receive prokinetic agents at least once, and that specific baseline patient characteristics, such as mechanical ventilation and abdominal surgery, would be associated with their use. Furthermore, we hypothesised that the use of prokinetic agents would not be associated with improved patient‐important outcomes [9].

## 2 Methods

### 2.1 Study Design and Setting

This international inception cohort study, conducted in 56 ICUs across 11 countries, was performed according to a pre‐published protocol [9] and overseen by a management committee (Tables S1 and S2).

Approval was obtained from the Danish Data Management Region of Zealand (EMN‐2024‐03449), and the study was registered with the Region of Zealand Centre for Data Compliance (p‐2024‐16493). Ethics approval was waived (EMN‐2024‐02746) in Denmark. All necessary regulatory approvals were obtained in participating countries (detailed in Table S3), and informed consent (written or verbal) was obtained where required.

Participating ICUs were recruited through an international investigator network within the established Collaboration for Research in Intensive Care (CRIC) [10]. Each ICU selected a 14‐day inception period between August 2024 and February 2025 (Table S4). Patients were followed for 90 days from index ICU admission.

This manuscript was prepared in accordance with the Strengthening the Reporting of Observational Studies in Epidemiology (STROBE) guideline (Table S5) [11].

### 2.2 Population

During the inception period, all acutely admitted, adult ICU patients (≥ 18 years) were eligible for inclusion. Patients transferred from other ICUs were included if they met the same criteria. Planned admissions (including planned post‐operative ICU stays), previously enrolled patients, and those declining informed consent if required were excluded. Patients requiring an unplanned acute ICU admission after elective surgery (e.g., due to complications) were included.

### 2.3 Data Collection and Management

All data were collected using an electronic case report form (eCRF) developed in the Research Electronic Data Capture (REDCap) system [12] hosted by the Region of Zealand, Denmark. Prior to study initiation, the eCRF was pilot‐tested (detailed in Table S6), and general information was distributed to participating sites.

Clinical data were extracted from medical records as predefined in the study protocol [9].

Baseline data included demographics, comorbidities (chronic pulmonary disease, history of severe heart failure, history of chronic liver failure, history of chronic renal failure, diabetes), selected treatments prior to ICU admission, and the Simplified Mortality Score for the Intensive Care Unit (SMS‐ICU) (Tables S7, S8a and S8b). The SMS‐ICU is an illness severity score ranging from 0 to 42 points, with higher scores indicating a higher risk of 90‐day mortality [13].

Daily data were collected throughout the ICU stay and resumed upon readmission to ICU or transfer to another participating ICU, for a maximum of 90 days. If a patient was transferred to a non‐participating ICU, data collection was discontinued. We recorded life‐support interventions (vasopressors, inotropes, mechanical ventilation and renal replacement therapy), SAEs (cardiac arrest, cardiac arrhythmias, extrapyramidal symptoms, severe diarrhoea and vomiting with clinically significant aspiration), enteral or oral intake (yes/no) and daily data on treatment with prokinetic agents (erythromycin, metoclopramide, domperidone or prucalopride). Information on prokinetic agents included dose, frequency, route of administration and duration of treatment. Detailed definitions are provided in Table S9.

On Day 90, vital status was assessed, and any additional admissions to non‐psychiatric hospitals were ascertained from medical records (Table S10).

### 2.4 Outcomes

The primary outcome was the proportion of patients who received prokinetic agents. Secondary outcomes were the number of patients with one or more SAEs, days alive without life support, days alive out of ICU, days alive out of hospital and all‐cause mortality, all assessed within 90 days (Table S11).

### 2.5 Statistical Methods

All analyses followed the pre‐published protocol and statistical analysis plan, with minor modifications, including modified admission groups, detailed in Table S12 [9].

#### 2.5.1 Sample Size

Based on prior studies, we assumed a prevalence of feeding intolerance of 25%, which we used as a proxy for the proportion of patients receiving prokinetic agents [2]. To estimate this proportion with a 95% confidence interval (CI) ranging from 22% to 28%, we planned a sample size of 1000 patients.

#### 2.5.2 Descriptive Data

Patients were stratified by any treatment with prokinetic agents during the ICU stay. We performed all analyses using R version 4.4.1 (R Core Team, R Foundation for Statistical Computing, Vienna, Austria).

Categorical variables are presented as counts with percentages, and continuous variables as medians with interquartile ranges (IQRs).

We summarised baseline characteristics, the occurrence of each SAE and other secondary outcomes descriptively and stratified by prokinetic treatment. Percentages are presented with 95% CIs calculated using the Clopper–Pearson method [14].

#### 2.5.3 Time to Treatment Analyses

Associations between baseline characteristics and time to first prokinetic agent use were assessed using pre‐specified Cox proportional hazards models, treating death and first ICU discharge as competing events; if a patient was discharged more than once, only the first discharge was considered.

The covariates assessed were ICU admission type (neurological condition, respiratory failure, circulatory failure, other), parenteral nutrition, number of comorbidities (1, 2, ≥ 3), mechanical ventilation, vasopressor/inotrope use, renal replacement therapy, abdominal surgery, emergency surgery and SMS‐ICU [15]. Covariates were selected a priori based on literature review, clinical relevance and the expected number of events [3, 16, 17].

#### 2.5.4 Association Between Prokinetic Treatment and SAEs or Mortality

We used time‐varying Cox proportional hazards models to evaluate the association between prokinetic treatment and SAEs, treating death and first ICU discharge as competing events. Prokinetic treatment was modelled as a time‐varying covariate, allowing for changes in treatment status daily during ICU stay.

We applied the same model to assess the association of prokinetic agents with mortality, using the first ICU discharge as the only competing event. All models were adjusted for country, SMS‐ICU, number of comorbidities, abdominal surgery, emergency surgery and ICU admission type.

Due to the low incidence of individual events, SAEs were analysed as a composite outcome.

#### 2.5.5 Regression Analyses of Secondary Outcomes

We analysed the association between treatment with prokinetic agents and secondary outcomes (days alive without life support, days alive out of ICU and days alive out of hospital) using linear regression models adjusted for predefined covariates [9]. In the primary analysis, deaths were assigned zero days. For patients transferred to a non‐participating ICU (*n* = 66), we used only observed days [18]. All days spent in participating ICUs, including readmissions, were counted. Secondary analyses explored best‐ and worst‐case scenarios for transfers and deaths (Table S13). Estimates were reported as mean differences (MDs) with 95% CIs and *p*‐values derived using non‐parametric bootstrapping with 50,000 resamples to obtain valid inference regardless of the distribution of data and model residuals.

#### 2.5.6 Assessment of Model Adequacy

Non‐linearity of continuous covariates was assessed for all models. For all Cox models, violations of the proportional hazards assumption [19] were handled using logarithmic time‐transformations (details in Table S14).

#### 2.5.7 Missing Data

We observed < 5% participants with missing data for one or more variables in the analyses (Table S15a and S15b). Therefore, all analyses were conducted using complete cases in line with the pre‐published statistical analysis plan [9]. Sixty‐six patients (4.6%) were transferred to non‐participating ICUs, resulting in missing daily variables. Losses to follow‐up and transfers led to censoring in the time‐to‐event analyses. For descriptive analyses, we assumed no events occurred during the time spent in the non‐participating ICUs (detailed in Table S15c).

#### 2.5.8 Significance and Interpretation

We interpreted two‐sided *p* < 0.05 or 95% CI not including 1 (for hazard ratios (HR)) or 0 (for MDs) as statistically significant. As pre‐specified [9], no adjustments were made for multiple comparisons.

## 3 Results

We included 1442 patients from 56 ICUs across 52 centres in 11 countries. Two patients were excluded, one who withdrew consent and one due to a lack of approval for data use. The final cohort comprised 1440 patients (Supporting Information S16, Figure S1). Most study sites were mixed ICUs (89%) in general hospitals (54%) (Table S17).

### 3.1 Baseline and ICU Admission Characteristics

Overall, 56.9% were male, the median age was 64 years (IQR 49–75), and 47.6% were admitted from emergency departments. Circulatory failure was the most common reason for ICU admission (26.1%), and 4.3% of patients were receiving prokinetic agents prior to ICU admission. Patients treated with prokinetic agents were more often admitted from general wards (38.0%), were younger (median age 61 years) and had higher illness severity at ICU admission (Table 1).

**TABLE 1:** Baseline characteristics.

	Overall, *N* = 1440	Prokinetic use^a^	
		No, *N* = 1253	Yes, *N* = 187
Age (years)	64 (49–75)	65 (50–75)	61 (48–73)
Male sex	819 (56.9%)	716 (57.1%)	103 (55.1%)
Comorbidities			
Pulmonary disease	275 (19%)	236 (18.8%)	39 (20.9%)
IHD or HF	267 (19.1%)	237 (18.9%)	30 (16.0%)
Chronic renal failure	135 (9.4%)	112 (9.0%)	23 (12.3%)
Chronic liver failure	68 (4.7%)	54 (4.3%)	14 (7.5%)
Diabetes (type 1 or 2)	389 (27.1%)	339 (27.1%)	50 (26.7%)
Haematologic malignancy or metastatic cancer	127 (8.8%)	107 (8.6%)	20 (10.7%)
Admitted from			
ED	686 (47.6%)	617 (49.2%)	69 (36.9%)
General ward	403 (28.0%)	332 (26.5%)	71 (38.0%)
OR	269 (18.7%)	233 (18.6%)	36 (19.3%)
ICU	82 (5.7%)	71 (5.7%)	11 (5.9%)
Surgery during hospital admission			
Elective surgery	174 (12.1%)	139 (11.1%)	35 (18.7%)
Emergency surgery	356 (24.8%)	307 (24.5%)	49 (26.2%)
Abdominal surgery	188 (13.1%)	152 (12.2%)	36 (19.3%)
Surgery before hospital admission			
Bariatric surgery	14 (1.0%)	13 (1.0%)	1 (0.5%)
Illness severity			
SMS‐ICU^b^	17 (13–22)	17 (13–22)	18 (13–22)
SMS‐ICU predicted 90%‐day mortality (%)^c^	25.3 (16.5–40.1)	25.3 (16.5–40.1)	28 (16.5–40.1)
Main reason for ICU admission			
Neurological condition	286 (19.9%)	242 (19.3%)	44 (23.5%)
Respiratory failure	359 (24.8%)	314 (24.9%)	45 (24.1%)
Circulatory failure	376 (26.1%)	327 (26.1%)	49 (26.2%)
Renal failure	51 (3.5%)	46 (3.7%)	5 (2.7%)
Liver failure	23 (1.6%)	17 (1.4%)	6 (3.2%)
GI bleeding	38 (2.6%)	34 (2.7%)	4 (2.1)
Other	307 (21.3%)	273 (21.8%)	34 (18.2%)
Treatments prior to ICU admission			
Prokinetic agents	62 (4.3%)	35 (2.8%)	27 (14.4%)
Parenteral nutrition^d^	62 (4.3%)	49 (3.9%)	13 (7.0%)
Country			
Denmark	641 (45%)	583 (47%)	58 (31%)
Saudi Arabia	193 (13%)	162 (13%)	31 (17%)
Sweden	114 (7.9%)	110 (8.8%)	4 (2.1%)
Finland	95 (6.6%)	81 (6.5%)	14 (7.5%)
Poland	90 (6.3%)	78 (6.2%)	12 (6.4%)
United Kingdom	75 (5.2%)	64 (5.1%)	11 (5.9%)
Spain	70 (4.9%)	39 (3.1%)	31 (17%)
Estonia	63 (4.4%)	49 (3.9%)	14 (7.5%)
Netherlands	55 (3.8%)	46 (3.7%)	9 (4.8%)
Kuwait	30 (2.1%)	29 (2.3%)	1 (0.5%)
Switzerland	14 (1.0%)	12 (1.0%)	2 (1.1%)

*Note:* Missing baseline data (seven patients, 0.5% in total): blood pressure: two patients (0.1%); information on elective surgery: one patient (0.1%); information on one comorbidity: three patients (0.2%); information on parenteral nutrition: one patient (0.1%) (detailed in Table S15a).

Continuous variables are presented as medians with interquartile range (IQR) and categorical variables as counts and percentages. Definitions of baseline variables are available in Table S8b.

Abbreviations: ED, emergency department; GI, gastrointestinal; HF, heart failure; ICU, intensive care unit; IHD, ischaemic heart disease; OR, operating or recovery room.

^a^ Treatment with erythromycin, metoclopramide, domperidone or prucalopride.

^b^ Median raw scores (IQR).

^c^ Predicted 90‐day mortality based on Simplified Mortality Score for the Intensive Care Unit (SMS‐ICU) (an illness severity score ranging from 0 to 42, details in Table S7).

^d^ Treatment with parenteral nutrition at the time of ICU admission.

The median duration of the initial ICU stay was 3 days (IQR 2.0–7.0). Patients not treated with prokinetic agents had a median stay of 3 days (IQR 2.0–6.0), and those treated with prokinetic agents had a median stay of 9 days (IQR 5.0–19.5) (Supporting Information S18, Figure S2). Seventy‐six patients (5.3%) were readmitted to the ICU during the 90‐day follow‐up.

### 3.2 Primary Outcome and Use of Prokinetic Agents

In total, 187 out of 1440 patients (13.0% 95% CI 11.3%–14.8%) received prokinetic agents during their ICU stay. Metoclopramide was the most frequently used agent (64.7%), followed by erythromycin (17.6%) (Table 2).

**TABLE 2:** Relative proportion of patients receiving specific prokinetic agents.

Prokinetic agent	Number of patients *n* = 187	% (95% CI)
Metoclopramide	121	64.7 (57.4–71.5)
Erythromycin	33	17.6 (12.5–23.9)
Domperidone	1	0.5 (0.0–2.9)
Prucalopride	0	0.0
Combination therapy^a^	27	14.4 (9.7–20.3)
Multiple agents (different days)^b^	5	2.7 (0.9–6.1)

*Note:* Proportions are based on the total number of patients who received prokinetic agents during their intensive care unit stay.

Abbreviation: CI, confidence interval.

^a^ Twenty‐seven (14.4%) patients received erythromycin and metoclopramide concomitantly.

^b^ Four patients (2.1%) received both metoclopramide and erythromycin on different days, and one patient (0.5%) received both metoclopramide and domperidone on separate days. Patients who received more than one agent are reported only in these two groups to avoid double counting.

Administered doses of metoclopramide ranged from 5 to 20 mg. The most common regimen was 10 mg three times daily, received by 61.4% (94/153) (S19, Figures S3 and S4). Of patients receiving metoclopramide, 51.0% (78/153) received it for 1 day, and 15% (23/153) for three consecutive days. The median treatment duration of the first treatment episode was 1 day (IQR 1–3 days).

For erythromycin, administered doses ranged from 100 to 500 mg. The most frequent regimen was 100 mg three times daily, administered to 32.8% (21/64). The most common treatment duration was two consecutive days (25.0%, 16/64) (Supporting Information S19, Figures S5 and S6) with a median of 3 days (IQR 2–4 days). Two patients received domperidone, and none received prucalopride.

Among patients treated with prokinetic agents, 27/187 (14.4%, 95% CI 9.7%–20.3%) received combination therapy with erythromycin and metoclopramide. No other combinations were observed. The most common combination was 100 mg of erythromycin and 10 mg of metoclopramide, both administered three times daily for a median of 3 days (IQR, 2–5 days).

The time from ICU admission to initiation of treatment ranged from one to 33 days, with a median of 4 days (IQR, 2–6) (Supporting Information S20, Figure S7).

Use of prokinetic agents varied considerably between countries (Table 1), ranging from 3.3% to 44.3% of patients. The highest proportions were reported in Spain (31/70; 44.3%) and Estonia (14/63; 22.2%), whereas the lowest were in Kuwait (1/30; 3.3%) and Sweden (4/113; 3.5%). In other countries, usage ranged from 9.0% to 16.4%. Metoclopramide was the predominant agent in all countries except Denmark and the Netherlands (Supporting Information S21, Figure S8).

### 3.3 Predictors of Prokinetic Agent Use

At baseline, patients who received prokinetic agents during their ICU stay were more likely to have undergone surgery and more frequently received parenteral nutrition at ICU admission (Table 1).

In both crude and adjusted analyses, abdominal surgery before ICU admission (adjusted HR 1.8, 95% CI 1.2–2.9) and admission in Spain (adjusted HR 3.5, 95% CI 2.2–5.6) were associated with starting prokinetic agents. No other baseline characteristics were statistically significantly associated with the use of prokinetic agents (Table 3).

**TABLE 3:** Baseline characteristics and time to prokinetic use.

Variable	Crude HR [95% CI]	*p*	Adjusted HR^a^ [95% CI]	*p*
SMS‐ICU^b^	1.00 [0.98, 1.02]	0.778	0.99 [0.96, 1.02]	0.596
1 comorbidity	1.08 [0.78, 1.50]	0.642	1.10 [0.78, 1.54]	0.591
2 comorbidities	0.91 [0.58, 1.42]	0.666	0.93 [0.58, 1.49]	0.768
≥ 3 comorbidities	1.11 [0.62, 1.99]	0.724	1.10 [0.59, 2.03]	0.767
Respiratory support	1.09 [0.80, 1.48]	0.590	1.04 [0.71, 1.53]	0.824
Use of inotropes	1.06 [0.79, 1.42]	0.708	1.16 [0.77, 1.73]	0.473
Renal replacement therapy	0.94 [0.56, 1.57]	0.818	1.00 [0.57, 1.75]	0.999
Parenteral nutrition	1.25 [0.71, 2.20]	0.438	1.05 [0.58, 1.89]	0.875
Admission reason^c^				
Respiratory failure	0.77 [0.51, 1.17]	0.219	0.80 [0.51, 1.23]	0.307
Circulatory failure	0.96 [0.64, 1.44]	0.841	0.91 [0.58, 1.43]	0.685
Other^d^	0.95 [0.63, 1.42]	0.791	0.85 [0.55, 1.32]	0.464
Abdominal surgery	1.53 [1.07, 2.21]	0.021	1.81 [1.17, 2.79]	0.008
Emergency surgery	1.07 [0.77, 1.48]	0.688	0.95 [0.63, 1.42]	0.797
Country^e^				
Switzerland	1.49 [0.36, 6.12]	0.576	1.58 [0.38, 6.59]	0.533
Estonia	1.76 [0.98, 3.16]	0.058	1.63 [0.90, 2.96]	0.105
Spain	3.25 [2.10, 5.05]	< 0.001	3.50 [2.19, 5.61]	< 0.001
Finland	1.72 [0.96, 3.08]	0.070	1.65 [0.91, 2.97]	0.096
Saudi Arabia	1.29 [0.83, 1.99]	0.261	1.41 [0.88, 2.25]	0.150
Kuwait	0.39 [0.05, 2.80]	0.348	0.38 [0.05, 2.82]	0.347
Netherlands	1.47 [0.73, 2.97]	0.282	1.55 [0.76, 3.17]	0.227
Poland	0.92 [0.49, 1.71]	0.782	0.88 [0.46, 1.68]	0.698
Sweden	0.53 [0.19, 1.47]	0.226	0.56 [0.20, 1.55]	0.263
United Kingdom	1.15 [0.60, 2.19]	0.678	1.17 [0.61, 2.27]	0.638

*Note:* Included in the analysis *n* = 1437.

Abbreviations: GI, gastrointestinal; HR, hazard ratio; SMS‐ICU, simplified mortality score for the intensive care unit.

^a^ Adjusted for SMS‐ICU, number of comorbidities (1, 2, ≥ 3), life support (mechanical ventilation (Y/N), vasopressor (Y/N) or renal replacement therapy (Y/N)), parenteral nutrition (Y/N), abdominal surgery (Y/N), emergency surgery (Y/N), admission reason (neurological, respiratory, circulatory, other) and country.

^b^ HR reflects the effect for a one‐point increase in SMS‐ICU: an illness severity score ranging from 0 to 42 points with higher scores indicating higher predicted 90‐day mortality.

^c^ Intensive care unit admission due to neurological condition was used as the reference category.

^d^ Other includes the following admission reason groups: renal failure, liver failure, GI bleeding and other.

^e^ Denmark served as the reference category for country as it included the largest number of patients.

### 3.4 Serious Adverse Events

**FIGURE 1:**
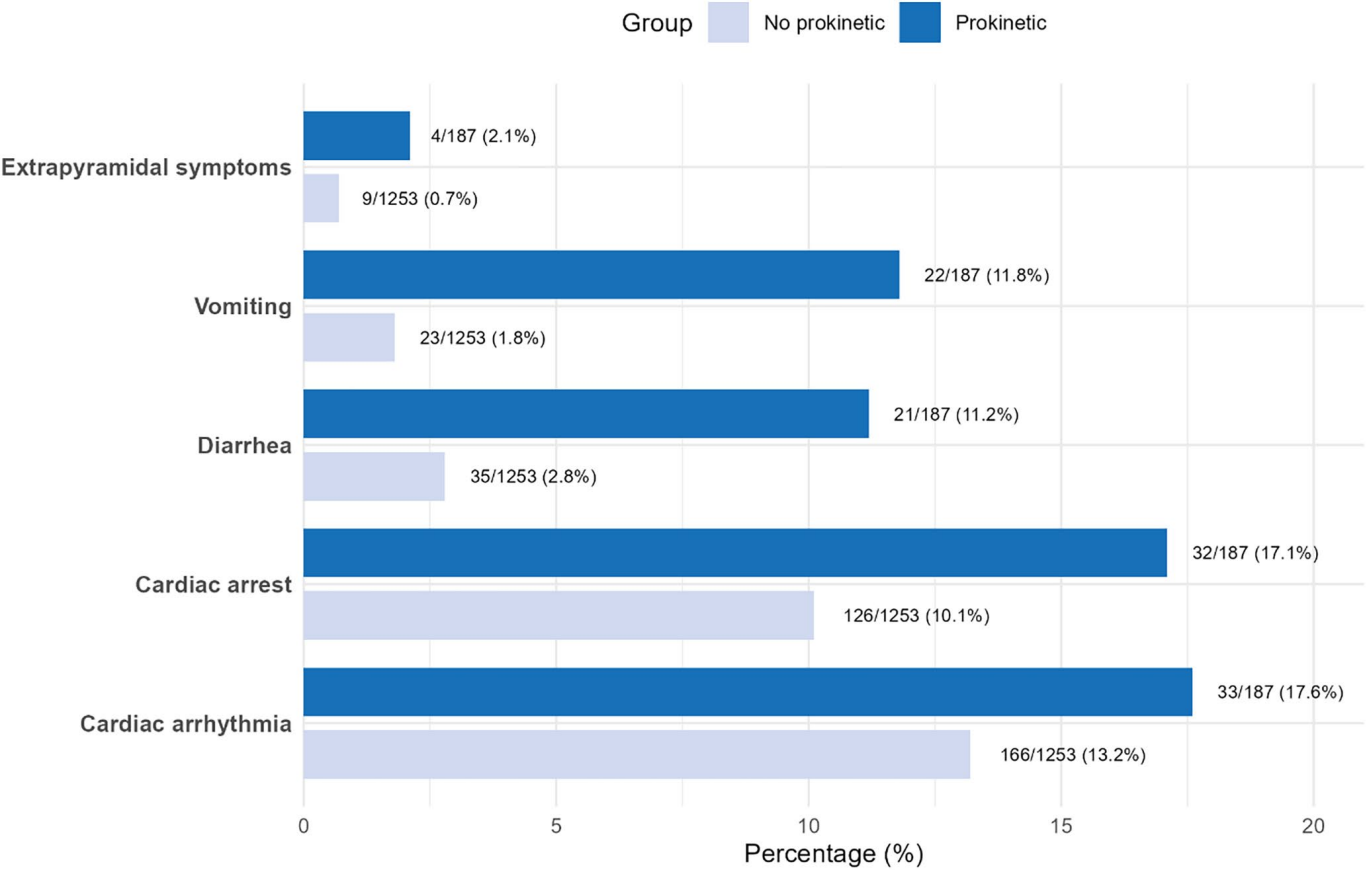
Proportion of patients experiencing serious adverse events. *N* = 1440, two patients (0.1%) had missing data for one serious adverse event (SAE) on a single intensive care unit (ICU) day but were included in the overview (Table S15b). The figure shows the proportion of patients experiencing each serious adverse event (SAE) stratified by prokinetic treatment (regardless of timing). Overall, 354 (24.6%) patients experienced one or more SAE, 277/1251 (22.1%) in the non‐treatment group and 77/187 (41.2%) in the treatment group. Percentages are calculated within each group. Patients may appear in multiple SAE categories but are counted only once per SAE type. Detailed SAE definitions are provided in Table S9.

Overall, 354/1440 patients (24.6%) experienced at least one SAE during ICU stay. The proportion was 77/187 (41.2%) among patients receiving prokinetic agents at any time during admission (irrespective of timing relative to the SAE) and 277/1253 (22.1%) among those not receiving prokinetic agents (Figure 1). Among patients who received prokinetic agents and experienced at least one SAE, 42.9% received the treatment before the first SAE.

The use of prokinetic agents was statistically significantly associated with a higher hazard of experiencing a SAE (HR 1.9, 95% CI 1.3–2.8, *p* < 0.001).

### 3.5 Mortality at 90 Days

Overall, 90‐day mortality was 367/1439 (25.5%, 95% CI 23.3–27.9), with 49/186 (26.3%, 95% CI 20.3–33.4) in patients who received prokinetic agents and 318/1253 (25.4%, 95% CI 23.0–27.9) in those who did not.

The use of prokinetic agents was not statistically significantly associated with the hazard of death (HR 0.6, 95% CI 0.3–1.2, *p* = 0.2).

### 3.6 Other Secondary Outcomes

Patients who received prokinetic agents had a lower median number of days alive and out of ICU (73 days [IQR 0–82] vs. 85 days [IQR 0–88], adjusted MD −7.6 days; 95% CI –13.4 to −2.2) and out of hospital (34 days [IQR 0–65] vs. 69 days [IQR 0–81], adjusted MD –13.5 days; 95% CI –18.7 to −8.4) (Table 4).

**TABLE 4:** Secondary outcomes stratified by prokinetic use.

Secondary outcomes	Overall (*n* = 1436), median (IQR)	No prokinetic use (*n* = 1250), median (IQR)	Prokinetic use (*n* = 186), median (IQR)	Adjusted MD (95% CI)	*p*
Days alive without life support	87 (0–89)	88 (0–90)	80 (0–86)	−5.51 days (−11.57 to 0.17)	0.067
Days alive out of ICU	84 (0–88)	85 (0–88)	73 (0–82)	−7.63 days (−13.41 to −2.12)	0.008
Days alive out of hospital	65 (0–80)	69 (0–81)	34 (0–65)	−13.54 days (−18.70 to −8.39)	< 0.001

*Note:* Data are presented as medians with interquartile range (IQR) for the 90‐day follow‐up period. Four patients (0.3%) were lost to follow‐up. Patients who died were assigned 0 days; only observed days were included for patients transferred to non‐participating ICUs (secondary analyses are presented in Table S23d).

Adjusted MDs account for SMS‐ICU, number of comorbidities, admission reason, abdominal surgery, emergency surgery and country, with estimates derived using non‐parametric bootstrapping (50,000 resamples).

Abbreviations: CI, confidence interval; ICU, intensive care unit; IQR, interquartile range; MD, mean differences.

Use of vasopressors, mechanical ventilation and renal replacement therapy was more frequent among patients treated with prokinetic agents (Table S22).

Median days alive without life support was 88 days (IQR 0–90) in patients not receiving prokinetic agents and 80 days (IQR 0–86) in those who did, with no statistically significant difference after adjustment (MD –5.5 days; 95% CI –11.6 to 0.2) (Table 4). Secondary analyses yielded similar results (Tables S23a–S23d).

## 4 Discussion

In this prospective international cohort study of 1440 acutely admitted adult ICU patients, 13.0% received prokinetic agents during their ICU stay, with metoclopramide being the most commonly used. We identified no statistically significant association between the use of prokinetic agents and the hazard of death, although CIs were wide and compatible with both reductions and increases. Use of prokinetic agents was more common after abdominal surgery and was associated with longer ICU and hospital stays, as well as a higher hazard of experiencing a SAE. These associations are likely influenced by confounding, including confounding by indication, as patients receiving prokinetic agents appeared more severely ill.

The proportion of patients receiving prokinetic agents was lower than hypothesised and lower than reported in previous studies [2, 20, 21]. In a 2015 retrospective observational study of 1888 ICU patients assessing the incidence of feeding intolerance, 37.9% received a prokinetic agent [21]. We hypothesised that the prevalence of feeding intolerance would correspond to the use of prokinetic agents; however, prevalence estimates vary widely by definition and may not directly reflect treatment patterns [2]. The lower proportion observed in our cohort is likely explained by variability in definitions of feeding intolerance, changes in feeding strategies [22] and shifts in prescribing practices over time.

We found variation in the use of prokinetic agents across countries, with prevalences ranging from 3% to 44%. These differences may reflect variations in clinical indications and the absence of standardised guidelines for managing gastrointestinal dysfunction in ICU patients [23]. Additionally, practice variation may be influenced by local guidelines, traditions and individual clinician preferences, as suggested by a recent survey of 830 ICU doctors reporting variations in symptoms used to assess feeding intolerance and in prokinetic use [24].

In our cohort, metoclopramide was the most frequently administered prokinetic agent despite European Society for Clinical Nutrition and Metabolism (ESPEN) guidelines recommending erythromycin as first‐line therapy [8]. This recommendation was based on a meta‐analysis of six randomised clinical trials (673 critically ill patients) comparing erythromycin or metoclopramide with placebo, which found a statistically significant improvement in feeding tolerance with erythromycin, but not with metoclopramide [8]. In addition, safety concerns, particularly the risk of neurological adverse effects, led United States and European regulatory agencies to restrict the use of metoclopramide to short‐term use (≤ 5 days) [25, 26].

The continued predominant use of metoclopramide in our cohort may be explained by the limited pharmacological alternatives for enhancing gastric emptying and concerns about the potential adverse effects of erythromycin, such as cardiac arrhythmias, as well as restrictions in some countries due to concerns about antimicrobial resistance [25, 27, 28, 29].

In our cohort, prokinetic agents were most often administered for 1–3 days, which aligns with a 2007 randomised trial in 90 mechanically ventilated critically ill patients with feeding intolerance that compared metoclopramide and erythromycin and showed a marked decline in efficacy for both after 3 days (to 27% for metoclopramide and 47% for erythromycin) [30]. This is also in line with ESPEN guidelines, which recommend limited use to 3 days due to reduced effectiveness beyond 72 h [8].

Use of prokinetic agents was associated with fewer days alive out of ICU and with a higher hazard of experiencing a SAE. This likely reflects residual confounding and the increased exposure time associated with longer ICU stays. Gastrointestinal dysfunction may both reflect and contribute to illness severity and is associated with increased morbidity and mortality in critical illness. Proposed mechanisms include gastrointestinal failure and increased intestinal permeability, which may act as early drivers of multi‐organ failure [31]. Hence, patients who are more severely ill are more likely to have gastrointestinal dysfunction, receive prokinetic agents and experience worse outcomes, independent of treatment effects [27]. Among patients who received prokinetic agents during ICU stay, 14% had started treatment at admission, and 7% were receiving parenteral nutrition, suggesting GI dysfunction already present on ICU arrival. Nonetheless, treatment effects cannot be ruled out.

Patients who underwent abdominal surgery prior to ICU admission were more likely to receive prokinetic agents, likely reflecting postoperative gastrointestinal dysfunction. Yet, a 2010 Cochrane systematic review of 39 randomised trials in patients undergoing abdominal surgery, comparing different prokinetic agents with placebo or no intervention, found insufficient evidence to support the use of metoclopramide and no effect of erythromycin on gastrointestinal recovery after abdominal surgery [32].

The strengths of this study are its prospective design and adherence to a pre‐published protocol with few deviations [9]. The study includes a consecutive, multinational ICU population, enhancing external validity.

This study also holds some limitations. First, 4.6% of the patients were transferred to a non‐participating ICU, resulting in missing daily variables. Second, to facilitate data collection across sites, we focused on broad categories, and information on the clinical indication for initiating prokinetic agents, as well as details such as the type of cardiac arrhythmias, was not recorded. Third, although admission and discharge times were available, interventions such as life support and prokinetic use were recorded as having occurred on the corresponding calendar day, irrespective of whether they were provided for the entire 24‐h period. Finally, the relatively small number of patients treated with prokinetics limited the statistical precision and interpretability of the association analyses.

## 5 Conclusions

In this international inception cohort study, 13% of ICU patients received prokinetic agents during their ICU stay, with metoclopramide being the most commonly used. Prokinetic use was more common among patients who had undergone abdominal surgery prior to ICU admission. Treatment with prokinetic agents was associated with fewer days alive outside the ICU and hospital at 90 days and a higher hazard of experiencing a SAE.

## Author Contributions

Conceptualisation: Vera Crone, Morten Hylander Møller, Anders Granholm, Anders Perner, Waleed Alhazzani, Laura Rindom Krogsgaard and Mette Krag. Methodology: Vera Crone, Anders Granholm, Morten Hylander Møller and Mette Krag. Data curation: Vera Crone. Formal analysis: Vera Crone and Anders Granholm. Investigation: All authors contributed to the investigation. Project administration: Vera Crone, Morten Hylander Møller and Mette Krag. Visualisation: Vera Crone, Morten Hylander Møller, Anders Granholm and Mette Krag. Writing – original draft: Vera Crone with input from Morten Hylander Møller, Anders Granholm and Mette Krag. Writing – review and editing: All authors.

## Funding

The authors have nothing to report.

## Conflicts of Interest

The Department of Intensive Care at Rigshospitalet—Copenhagen University Hospital (M.H.M., A.G., A.P.) has received funding from the Independent Research Fund Denmark, Novo Nordisk Foundation and Sygeforsikringen ‘danmark’ outside the submitted work. A.R.B. is holding a grant from the Estonian Research Council (PRG1255). J.H. has received reimbursement for advisory board work (Paion) outside the submitted work. Ricard Ferrer has received honoraria from Shionogi MSD Gilead Menarini Thermofisher Viatris AOP outside the submitted work. M.O. has received research funding from Baxter and Biomerieux (paid to institution). The Department of Intensive Care at Copenhagen University Hospital—North Zealand (MHB) has received funding for other research activities from Sygeforsikring ‘danmark’ and Svend Andersen Foundation. M.H.B. has received reimbursement for advisory board work from Biomerieux outside the submitted work.

## Supporting information


**Table S1:** Management committee.
**Table S2:** Participating countries and national investigators.
**Table S3:** Ethics.
**Table S4:** Participating sites and investigators.
**Table S5:** Strengthening the reporting of observational studies in epidemiology (STROBE) checklist.
**Table S6:** Pilot‐test of the eCRF prior to study initiation.
**Table S7:** Simplified Mortality Score for the Intensive Care Unit (SMS‐ICU).
**Table S8:** Baseline variables and definitions.
**Table S9:** Daily variables and definitions.
**Table S10:** Follow‐up variables and definitions.
**Table S11:** Outcome measures.
**Table S12:** Deviations from the protocol.
**Table S13:** Best and worst case scenarios.
**Table S14:** Model diagnostics and assumption checks.
**Table S15:** Missing data.
**S16**. Study flow diagram.
**Table S17:** Site characteristics.
**S18**. Length of ICU stay.
**S19**. Data on prokinetics.
**S20**. Time to first prokinetic treatment.
**S21**. Prokinetic use by country.
**Table S22:** Use of life support.
**Table S23:** Descriptive overview and regression analyses of secondary outcomes.
**Figure S1:** Only patients meeting all inclusion criteria were screened for inclusion.
**Figure S2:** Length of ICU stay during first (index) ICU admission stratified by prokinetic treatment.
**Figure S3:** Metoclopramide dose‐frequency combinations among treated patients (*n* = 153, 81.8%).
**Figure S4:** Consecutive days of the first metoclopramide treatment episode (*n* = 153, 81.8%).
**Figure S5:** Erythromycin dose‐frequency combinations among treated patients (*n* = 64, 34.2%).
**Figure S6:** Consecutive days of the first erythromycin treatment episode.
**Figure S7:** Time to first prokinetic treatment during index ICU admission (*n* = 179, 95.7%).
**Figure S8:** Prokinetic treatment patterns by country.

## Data Availability

Anonymised data supporting the findings of this study can be made available from the corresponding author (V.C.) upon reasonable request, subject to applicable permissions and data sharing agreements.
