# Supplementary material for: Use of Prokinetic Agents in Adult ICU Patients: An International Inception Cohort Study (PATIENCE)
Source: Acta Anaesthesiol Scand. 2026 Jun 24;70(7):e70290. doi: 10.1111/aas.70290 (PMC13291764; doi:10.1111/aas.70290)
Supplement: Supplementary file 1 — Table S1: Management committee. Table S2: Participating countries and national investigators. Table S3: Ethics. Table S4: Participating sites and investigators. Table S5: Strengthening the reporting of observational studies in epidemiology (STROBE) checklist. Table S6: Pilot‐test of the eCRF prior to study initiation. Table S7: Simplified Mortality Score for the Intensive Care Unit (SMS‐ICU). Table S8: Baseline variables and definitions. Table S9: Daily variables and definitions. Table S10: Follow‐up variables and definitions. Table S11: Outcome measures. Table S12: Deviations from the protocol. Table S13: Best and worst case scenarios. Table S14: Model diagnostics and assumption checks. Table S15: Missing data. S16. Study flow diagram. Table S17: Site characteristics. S18. Length of ICU stay. S19. Data on prokinetics. S20. Time to first prokinetic treatment. S21. Prokinetic use by country. Table S22: Use of life support. Table S23: Descriptive overview and regression analyses of secondary outcomes. Figure S1: Only patients meeting all inclusion criteria were screened for inclusion. Figure S2: Length of ICU stay during first (index) ICU admission stratified by prokinetic treatment. Figure S3: Metoclopramide dose‐frequency combinations among treated patients (n = 153, 81.8%). Figure S4: Consecutive days of the first metoclopramide treatment episode (n = 153, 81.8%). Figure S5: Erythromycin dose‐frequency combinations among treated patients (n = 64, 34.2%). Figure S6: Consecutive days of the first erythromycin treatment episode. Figure S7: Time to first prokinetic treatment during index ICU admission (n = 179, 95.7%). Figure S8: Prokinetic treatment patterns by country. [file AAS-70-0-s001.docx]

**SUPPLEMENTARY MATERIAL**

**Supplement to:**

**Use of prokinetic agents in adult ICU patients: an international inception cohort study (PATIENCE)**

**Authors:** Vera Crone^1^, Morten Hylander Møller^2,3^, Anders Granholm^2,4^, Anders Perner^2,3^, Waleed Alhazzani^5,6^, Laura Rindom Krogsgaard^7^, Abdulrahman Al-Fares^8^, Johanna Hästbacka^9^, Marlies Ostermann^10^, Carmen A Pfortmueller^11^, Ricard Ferrer^12^, Annika Reintam Blaser^13,14^, Olof Wall^15^, Eric Keus^16^, Wojciech Szczeklik^17^, Peter Martin Hansen^18^ , Jens Michelsen^19^, Mikkel Bak^19^, Lise Skyttegaard Balkert^19^, Tino Severinsen^20^, Anne Craveiro Brøchner^21,22^, Jonas Elmer Pedersen^23^, Anne Sofie Andreasen^24^, Camilla Tageby Nielsen^24^, Theis Skovsgaard Itenov^25,3^, Maj-Brit Nørregaard Kjær^2^, Zemira Engbakken^2^, Morten H. Bestle^26,2^, Peter Buhl Hjortrup^27^, Christian Juhl Svendsen^28^, Sandra Lindkvist-Viggers^29^, Björn Anders Brand^30^, Andreas Bender Jonsson^31^, Tobias Browall Krogh^32^, Kristian Reinhold Jauho^33^, Mathias Sinkbæk Thomsen^34^, Therese Simonsen Straarup^35^, Eva Poulsen^35^, Christian Gade Nissen^36^, Olena Breum^36^, Per Lund Petersen^37^, Christopher Torp^38^, Bodil Steen Rasmussen^39,40^, Anne-Marie Gellert Bunzel^39^, Seraina-Flavia Buholzer^11^, Elizabeth Hardman^10^, Daniel Törnberg^15,41^, David S. Pérez^12^, Kenneth van Smaalen^16^, Marisa Onrust^16^, Liivi Maddison^42^, Oskar Appelberg^42^, Hans-Erik Ehrlich^42^, Kadri Tamme^43,13^, Reile Juhanson^43^, Jaanus Korjas^44^, Ville Jalkanen^9^, Marleena Finni^45^, Matti Reinikainen^46^, Talvikki Koskue^47^, Marcelina Czok^17^, Pawel Zatorski^48^, Oliwia Doroba^49^, Bartosz Kudlinski^50^, Pawel Nowak^50^, Szymon Bialka^51^, Anna Kluzik^52^, Hanna Baran^53^, Zainab Al Duhailib^54,55^, Hakeam A. Hakeam^54,56^, Mohammed Alshahrani^57^, Osama Ahmed Elfaki^58^, Fadah A. Alanazi^59^, Rakan Aldhahri^60^, Khairallah Belkhouja^60^, Ali Alahmari^61^, Rawabi M. Alsayer ^5,62^, Ola Friman^63^, Anca Balintescu^64^ and Mette Krag ^3,65^

**Corresponding author:**

**Vera Crone, MD**

**Department of intensive care, Holbæk Hospital, Holbæk, Denmark**

**veracrone@gmail.com**

Table of contents

[S 1 - Management committee 3](#_Toc223121555)

[S 2 - Participating countries and national investigators 3](#_Toc223121556)

[S 3 – Ethics 4](#_Toc223121557)

[S 4 - Participating sites and investigators 5](#_Toc223121558)

[S 5 - Strengthening the reporting of observational studies in epidemiology (STROBE) checklist 9](#_Toc223121559)

[S 6 - Pilot-test of the eCRF prior to study initiation 12](#_Toc223121560)

[S 7 - Simplified Mortality Score for the Intensive Care Unit (SMS-ICU) 13](#_Toc223121561)

[S 8 - Baseline variables and definitions 14](#_Toc223121562)

[S 9 - Daily variables and definitions 17](#_Toc223121563)

[S 10 - Follow-up variables and definitions 18](#_Toc223121564)

[S 11 - Outcome measures 19](#_Toc223121565)

[S 12 - Deviations from the protocol 20](#_Toc223121566)

[S 13 – Best and worst case scenarios 21](#_Toc223121567)

[S 14 - Model diagnostics and assumption checks 22](#_Toc223121568)

[S 15 - Missing data 22](#_Toc223121569)

[S 16 - Study flow diagram 24](#_Toc223121570)

[S 17 - Site characteristics 25](#_Toc223121571)

[S 18 - Length of ICU stay 26](#_Toc223121572)

[S 19 - Data on prokinetics 27](#_Toc223121573)

[S 20 - Time to first prokinetic treatment 30](#_Toc223121574)

[S 21 - Prokinetic use by country 31](#_Toc223121575)

[S 22 - Use of life support 32](#_Toc223121576)

[S 23 - Descriptive overview and regression analyses of secondary outcomes 33](#_Toc223121577)

[References 34](#_Toc223121578)

### S 1 - Management committee

Table S1

| Name | Affiliation |
| --- | --- |
| Vera Crone | Department of Intensive Care, Holbæk Hospital, Holbæk, Denmark^1^ |
| Morten Hylander Møller | Department of Intensive Care, Copenhagen University Hospital - Rigshospitalet, Copenhagen, Denmark and Department of Clinical Medicine, University of Copenhagen, Copenhagen, Denmark |
| Anders Granholm | Department of Intensive Care, Copenhagen University Hospital - Rigshospitalet, Copenhagen, Denmark and Section of Biostatistics, Department of Public Health, University of Copenhagen, Copenhagen, Denmark |
| Anders Perner | Department of Intensive Care, Copenhagen University Hospital - Rigshospitalet, Copenhagen, Denmark and Department of Clinical Medicine, University of Copenhagen, Copenhagen, Denmark |
| Waleed Alhazzani | Health Research Center of Excellence, Ministry of Defense Health Services, Riyadh, Saudi Arabia |
| Laura Rindom Krogsgaard | Gastrounit, Section of Gastroenterology, Hvidovre Hospital, Denmark |
| Mette Krag | Department of Anaesthesiology, Surgery and Trauma Centre, Copenhagen University Hospital – Rigshospitalet and Department of Clinical Medicine, University of Copenhagen, Copenhagen, Denmark |

*^1^ Coordinating centre*

### S 2 - Participating countries and national investigators

Table S2

| **National investigator** | **Country** | **Centre** |
| --- | --- | --- |
| Vera Crone (PI) | Denmark | Department of Intensive Care, Holbæk Hospital, Holbæk |
| Annika Reintam Blaser | Estonia | Department of Anaesthesiology and Intensive Care, University of Tartu, Tartu |
| Johanna Hästbacka | Finland | Department of Intensive Care, Tampere University Hospital, Wellbeing Services County of Pirkanmaa and Tampere University, Tampere |
| Abdulrahman Al-Fares | Kuwait | Department of Anaesthesia, Critical Care Medicine and Pain Medicine, Al-Amiri Hospital, Kuwait City |
| Eric Keus | Netherlands | Dept. of Critical Care, University Medical Center Groningen, University of Groningen, Groningen |
| Wojciech Szczeklik | Poland | Center for Intensive Care and Perioperative Medicine, Jagiellonian University Medical College, Krakow |
| Waleed Alhazzani  Rawabi M. Alsayer | Saudi Arabia | Health Research Center of Excellence, Ministry of Defense Health Services, Riyadh |
| Ricard Ferrer | Spain | Department of Intensive Care,Vall d'Hebron Hospital Universitari, Vall d'Hebron Barcelona, Hospital Campus, Barcelona |
| Olof Wall | Sweden | Department of Intensive Care, Danderyd Hospital, Stockholm |
| Carmen Pfortmueller | Switzerland | Department of Intensive Care, Inselspital, Bern University Hospital and University of Bern, Bern |
| Marlies Ostermann | The United Kingdom | Department of Critical Care, King's College London, Guy's and St. Thomas' NHS Foundation Trust, London |

*Abbreviation: Principal investigator (PI)*

### S 3 – Ethics

Table S3

Summary of ethics approvals across participating countries, data from Denmark is included in the main manuscript. The study complied with local ethical and regulatory requirements in all countries and informed consent was obtained in countries requiring it. Where applicable, data sharing agreements were established between Denmark and each participating country/site.

| **Country** | **Approvals** |
| --- | --- |
| Estonia | Ethics approval obtained |
| Finland | Ethics approval obtained |
| Kuwait | Ethics approval obtained |
| Netherlands | Ethics approval obtained |
| Poland | Ethics approval obtained |
| Saudi Arabia | Ethics approval obtained |
| Spain | Ethics approval obtained |
| Sweden | Ethics approval obtained |
| Switzerland | Ethics approval obtained |
| United Kingdom | Approved and conducted as a service evaluation |

### S 4 - Participating sites and investigators

Table S4

| **Site investigator(s) and contributor(s)** | **Country** | **Department** | **Inclusion period (both dates included)** |
| --- | --- | --- | --- |
| Peter Martin Hansen (SI) | Denmark | Department of Intensive Care, Odense University Hospital, Svendborg | 23-09-24 to 06-10-24 |
| Jens Michelsen (SI)  Mikkel Bak (SI)  Lise Skyttegaard Balkert (SI)  Emma Hertel Larsen  Rikke Graae Brandt-Hauritz | Denmark | Department of Intensive Care, Odense University Hospital, Odense  -Neurointensive Care Unit  -General Intensive Care Unit  -Thoracic Intensive Care Unit | 23-09-24 to 06-10-24 |
| Tino Severinsen (SI)  Thomas Strøm | Denmark | Department of Intensive Care, University Hospital of Southern Denmark, Aabenraa | 03-12-24 to 17-12-24 |
| Anne Brøchner (SI) | Denmark | Department of Anaesthesiology and Intensive Care, University Hospital of Southern Denmark, Kolding | 08-12-24 to 22-12-24 |
| Jonas Elmer Pedersen (SI)  Morten Rune Blichfeldt-Eckhardt | Denmark | Department of Anaesthesiology and Intensive Care, Lillebælt Hospital, Vejle | 09-09-24 to 22-09-24 |
| Anne Sofie Andreasen (SI)  Camilla Tageby Nielsen | Denmark | Department of Anaesthesiology and Intensive Care, Copenhagen University Hospital - Herlev-Gentofte Hospital, Herlev | 28-10-24 to 10-11-24 and 06-01-25 to 19-01-25 |
| Theis Itenov (SI)  Diana Bertelsen Jensen  Anna Marie Friis Pedersen | Denmark | Department of Anaesthesiology and Intensive Care, Copenhagen University Hospital - Bispebjerg and Frederiksberg Hospital, Copenhagen | 30-09-24 to13-10-24 |
| Maj-Brit Nørregaard Kjær (SI)  Jette Fredlund Degn  Kis Rønn Uhre  Anne Witte Kamstrup  Zemira Engbakken | Denmark | Department of Intensive Care, Copenhagen University Hospital - Rigshospitalet, Copenhagen | 30-09-24 to13-10-24 and 06-01-25 to 19-01-25 |
| Morten H. Bestle (SI)  Sanne Lauritzen | Denmark | Department of Anaesthesiology and Intensive Care, Copenhagen University Hospital -North Zealand, Hillerød | 16-09-24 to 29-09-24 |
| Peter Buhl Hjortrup (SI) | Denmark | Department of Cardiothoracic Anaesthesia and Intensive Care, Copenhagen University Hospital - Rigshospitalet, Copenhagen | 30-09-24 to13-10-24 |
| Christian Juhl Svendsen (SI) | Denmark | Department of Anaesthesiology and Intensive Care, Copenhagen University Hospital - Amager and Hvidovre, Hvidovre | 19-08-24 to 01-09-24 |
| Sandra Lindkvist-Viggers (SI) | Denmark | Department of Neuroanaesthesiology, Copenhagen University Hospital - Rigshospitalet, Copenhagen | 30-09-24 to13-10-24 |
| Björn Anders Brand (SI) | Denmark | Department of Anaesthesiology and Intensive Care, Copenhagen University Hospital - Herlev-Gentofte Hospital, Gentofte | 12-08-24 to 25-08-24 |
| Vera Crone (SI) | Denmark | Department of Anaesthesiology and Intensive Care, Holbæk Hospital, Holbæk | 19-08-24 to 01-09-24 |
| Andreas Bender Jonsson (SI) | Denmark | Department of Anaesthesiology and Intensive Care, Zealand University Hospital, Roskilde | 09-09-24 to 22-09-24 |
| Tobias Browall Krogh (SI) | Denmark | Department of Anaesthesiology and Intensive Care, Zealand University Hospital, Køge | 09-09-24 to 22-09-24 |
| Kristian Reinhold Jauho (SI) | Denmark | Department of Anaesthesiology and Intensive Care, Zealand University Hospital, Nykøbing Falster | 19-08-24 to 01-09-24 |
| Mathias Sinkbæk Thomsen (SI) Helle Bundgaard | Denmark | Department of Intensive Care, Randers Regional Hospital, Randers | 09-09-24 to 22-09-24 |
| Therese Simonsen Straarup (SI)  Eva Poulsen (SI) | Denmark | Department of Anaesthesiology and Intensive Care, Viborg Regional Hospital, Viborg | 09-09-24 to 22-09-24 and 06-01-25 to 19-01-25 |
| Christian Gade Nissen (SI) | Denmark | Department of Intensive Care (North), Aarhus University Hospital, Aarhus | 23-09-24 to 06-10-24 |
| Olena Breum (SI) | Denmark | Department of Intensive Care (East), Aarhus University Hospital, Aarhus | 16-09-24 to 29-09-24 |
| Christopher Torp (SI) | Denmark | Department of Intensive Care, Gødstrup Hospital, Gødstrup | 28-10-24 to 10-11-24 |
| Per Lund Petersen (SI)  Kjeld Asbjørn Jensen Damgaard | Denmark | Department of Intensive Care, North Denmark Regional Hospital – Hjørring | 30-09-24 to 03-11-24* |
| Bodil Steen Rasmussen (SI)  Anne-Marie Gellert Bunzel | Denmark | Department of Anaesthesiology and Intensive Care, Aalborg University Hospital, Aalborg University, Aalborg  -Neurointensive Care Unit  -General Intensive Care Unit  -Thoracic Intensive Care Unit | 28-10-24 to 10-11-24 |
| Liivi Maddison (SI)  Oskar Appelberg  Hans-Erik Ehrlich | Estonia | Intensive Care Centre, North Estonia Medical Centre, Tallinn | 02-12-24 to15-12-24 |
| Reile Juhanson (SI)  Kadri Tamme | Estonia | Department of Anaesthesiology and Intensive Care, Tartu University Hospital, Tartu | 02-12-24 to15-12-24 |
| Jaanus Korjas (SI)  Maarja Hallik  Priido Linntam | Estonia | Centre of Anaesthesiology and Intensive Care, East Tallinn Central Hospital, Tallinn | 02-12-24 to15-12-24 |
| Marleena Finni (SI)  Leena Pettilä  Minttu Saario  Elina Lappi  Jonna Heinonen | Finland | Intensive Care Units, Helsinki University Hospital, Helsinki, Finland | 27-01-25 to 09-02-24 |
| Matti Reinikainen (SI)  Marisanna Tavasti  Petra Kankkunen  Mikko Pietarinen | Finland | Intensive Care Unit, Kuopio University Hospital, Wellbeing Services County of North Savo, Kuopio, Finland | 17-02-25 to 02-03-25 |
| Talvikki Koskue (SI) | Finland | Intensive Care Unit, Päijät-Häme Central Hospital, Lahti, Finland | 20-01-25 to 02-02-25 |
| Helka Tervas (SI)  Annika Kivioja | Finland | Intensive Care Unit, Tampere University Hospital, Wellbeing Services County of Pirkanmaa, Tampere, Finland | 13-01-25 to 26-01-25 |
| Abdulrahman Al-Fares (SI) | Kuwait | Department of Anesthesia, Critical Care Medicine and Pain Medicine, Al-Amiri Hospital, Kuwait City, Kuwait. | 01-11-24 to 15-11-2024 |
| Kenneth van Smaalen (SI)  Marisa Onrust  Fredrike Zwiers-Blokzijl  Eric Keus | Netherlands | Dept. of Critical Care, University Medical Center Groningen, University of Groningen, Groningen, The Netherlands | 18-11-24 to 01-12-24 |
| Marcelina Czok (SI) | Poland | Center for Intensive Care and Perioperative Medicine, Jagiellonian University Medical College | 10-02-25 to 23-02-25 |
| Pawel Zatorski (SI) | Poland | First Department of Anesthesiology and Intensive Care Medical University of Warsaw | 17-02-24 to 02-03-25 |
| Oliwia Doroba (SI) | Poland | Second Department of Anesthesiology and Intensive Care Medical University of Warsaw | 10-02-25 to 23-02-25 |
| Bartosz Kudlinski (SI)  Pawel Nowak | Poland | Clinical Department of Anesthesiology and Intensive Care University of Zielona Góra Collegium Medicum | 10-02-25 to 23-02-25 |
| Szymon Bialka (SI) | Poland | Department of Anesthesiology and Intensive Care Independent Public Clinical Hospital No. 1 in Zabrze | 03-02-25 to 16-02-25 |
| Anna Kluzik (SI) | Poland | Clinical Department of Anesthesiology, Intensive Care and Pain Management University Clinical Hospital in Poznań | 10-02-25 to 23-02-25 |
| Hanna Baran (SI) | Poland | Clinic of Anesthesiology and Intensive Therapy,University Clinical Hospital No. 1 named after Tadeusz Sokołowski in Szczecin | 17-02-24 to 02-03-25 |
| Zainab Al Duhailib (SI)  Hakeam Hakeam (SI)  Munirah Alshalawi | Saudi Arabia | Critical Care Medicine Department, King Faisal Specialist Hospital and Research Centre, Riyadh, Saudi Arabia | 15-12-24 to 29-12-24 |
| Mohammed Alshahrani (SI)  Charlene Mapusao  Laila Perlas Asonto | Saudi Arabia | Critical Care Department, King Fahad University Hospital, Imam Abdulrahman Bin Faisal University, Dammam | 15-12-24 to 29-12-24 |
| Osama Ahmed Elfaki (SI)  Hanan Hassan Alsomali  Wejdan Ahmed Abuillah  Hassna Hussain AlAnazi  Maitha Mohammaed Albinali | Saudi Arabia | Department of Intensive Care, Prince Sultan Military Medical City, Riyadh | 15-12-24 to 29-12-24 |
| Abdulaziz Alshaer (SI)  Fadah A. Alanazi | Saudi Arabia | Department of Intensive Care, King Fahad Military Medical Complex, Dhahran | 02-01-24 to 16-01-25 |
| Khairallah Belkhouja (SI)  Rakan Aldhahri  Raed Alsaddi  Reem Alwasiyah  Maha Awaad Alqurashi | Saudi Arabia | Department of Intensive Care, King Fahad Armed Forces Hospital, Jeddah | 05-01-25 to 19-01-25 |
| Ali Alahmari (SI)  Shady Mohamed Abosamra  Odai Hatem Hejazi  Raeed Saeed Algarni | Saudi Arabia | Department of Intensive Care, Armed Forces Hospital, Southern Region, Khamis Mushayt | 06-01-25 to 19-01-25 |
| David Pérez (SI)  Jordi Canals Serrat  Vanessa Casares  Anna Casas Cubells | Spain | Department of Intensive Care, Vall d'Hebron Hospital Universitari, Vall d'Hebron Barcelona, Hospital Campus, Barcelona | 25-11-24 to 08-12-25 |
| Daniel Törnberg (SI)  Olof Wall (SI) | Sweden | Department of Anaesthesia and Intensive Care, Danderyd Hospital, Stockholm | 10-02-25 to23-02-25 |
| Ola Friman (SI)  Pia Zetterquist  Viveca Hamback-Hellkvist | Sweden | Department of Intensive Care, Karolinska University Hospital, Stockholm | 17-02-25 to 02-03-25 |
| Anca Balintescu (SI)  Elisabeth Hellgren  Fredrik Sjöberg | Sweden | Department of Clinical Science and Education, Section of Anaesthesia and Intensive Care, South General Hospital, Karolinska Institute, Stockholm | 10-02-25 to 23-02-25 |
| Seraina-Flavia Buholzer (SI)  Carmen Pfortmüller | Switzerland | Department of Intensive Care, Inselspital, Bern University Hospital and University of Bern, Bern | 13-08-24 to 25-08-24 |
| Elizabeth Hardman (SI)  Jayanta Das  Georgia Green  Lais Barroso  Iryna Melnyk  Megan Hollands  Ummara Shahid  Marlies Ostermann | United Kingdom | Department of Critical Care, King's College London, Guy's and St. Thomas' NHS Foundation Trust, London | 09-12-24 to 22-12-24 |

^*^*Extended due to few intensive care unit admissions during initial inclusion period*

*SI: Site Investigator*

### S 5 - Strengthening the reporting of observational studies in epidemiology (STROBE) checklist

Table S5

STROBE Statement—checklist of items that should be included in reports of cohort studies

|  | Item No. | Recommendation | Page  No. |  |
| --- | --- | --- | --- | --- |
| **Title and abstract** | 1 | (*a*) Indicate the study’s design with a commonly used term in the title or the abstract | 1 |  |
|  |  | (*b*) Provide in the abstract an informative and balanced summary of what was done and what was found | 5 |  |
| Introduction | | | |  |
| Background/rationale | 2 | Explain the scientific background and rationale for the investigation being reported | 6 |  |
| Objectives | 3 | State specific objectives, including any prespecified hypotheses | 6 |  |
| Methods | | | |  |
| Study design | 4 | Present key elements of study design early in the paper | 7-8 |  |
| Setting | 5 | Describe the setting, locations, and relevant dates, including periods of recruitment, exposure, follow-up, and data collection | 7-8 and Appendix |  |
| Participants | 6 | (*a*) Give the eligibility criteria, and the sources and methods of selection of participants. Describe methods of follow-up | 7-8 |  |
|  |  | (*b*) For matched studies, give matching criteria and number of exposed and unexposed | NA |  |
| Variables | 7 | Clearly define all outcomes, exposures, predictors, potential confounders, and effect modifiers. Give diagnostic criteria, if applicable | 7-8 + Appendix |  |
| Data sources/ measurement | 8* | For each variable of interest, give sources of data and details of methods of assessment (measurement).  Describe comparability of assessment methods if there is more than one group | 7-8 + Appendix  NA |  |
| Bias | 9 | Describe any efforts to address potential sources of bias | 9 |  |
| Study size | 10 | Explain how the study size was arrived at | 8 |  |

Continued on next page

| Quantitative variables | 11 | Explain how quantitative variables were handled in the analyses. If applicable, describe which groupings were chosen and why | 8 |  |
| --- | --- | --- | --- | --- |
| Statistical methods | 12 | (*a*) Describe all statistical methods, including those used to control for confounding | 9-10 + Appendix |  |
|  |  | (*b*) Describe any methods used to examine subgroups and interactions | 9-10 |  |
|  |  | (*c*) Explain how missing data were addressed | 10 |  |
|  |  | (*d*) If applicable, explain how loss to follow-up was addressed | 10 |  |
|  |  | (*e*) Describe any sensitivity analyses | Appendix |  |
| Results | | | |  |
| Participants | 13* | (a) Report numbers of individuals at each stage of study—eg numbers potentially eligible, examined for eligibility, confirmed eligible, included in the study, completing follow-up, and analysed | 11 + Appendix |  |
|  |  | (b) Give reasons for non-participation at each stage |  |  |
|  |  | (c) Consider use of a flow diagram | Appendix |  |
| Descriptive data | 14* | (a) Give characteristics of study participants (eg demographic, clinical, social) and information on exposures and potential confounders | 11 + table 1  + Appendix |  |
|  |  | (b) Indicate number of participants with missing data for each variable of interest | Appendix |  |
|  |  | (c) Summarise follow-up time (eg, average and total amount) | 12 |  |
| Outcome data | 15* | *Cohort study*—Report numbers of outcome events or summary measures over time | 11 + table 2 |  |
| Main results | 16 | (*a*) Give unadjusted estimates and, if applicable, confounder-adjusted estimates and their precision (eg, 95% confidence interval). Make clear which confounders were adjusted for and why they were included | 12-13 + Appendix |  |
|  |  | (*b*) Report category boundaries when continuous variables were categorized | - |  |
|  |  | (*c*) If relevant, consider translating estimates of relative risk into absolute risk for a meaningful time period | - |  |

Continued on next page

| Other analyses | 17 | Report other analyses done—eg analyses of subgroups and interactions, and sensitivity analyses | Appendix |  |
| --- | --- | --- | --- | --- |
| Discussion | | | | |
| Key results | 18 | Summarise key results with reference to study objectives | 14-15 |  |
| Limitations | 19 | Discuss limitations of the study, taking into account sources of potential bias or imprecision. Discuss both direction and magnitude of any potential bias | 15-16 |  |
| Interpretation | 20 | Give a cautious overall interpretation of results considering objectives, limitations, multiplicity of analyses, results from similar studies, and other relevant evidence | 14-16 |  |
| Generalisability | 21 | Discuss the generalisability (external validity) of the study results | 15 |  |
| Other information | |  | | |
| Funding | 22 | Give the source of funding and the role of the funders for the present study and, if applicable, for the original study on which the present article is based | 3 |  |

*Give information separately for cases and controls in case-control studies and, if applicable, for exposed and unexposed groups in cohort and cross-sectional studies.

**Note:** An Explanation and Elaboration article discusses each checklist item and gives methodological background and published examples of transparent reporting. The STROBE checklist is best used in conjunction with this article (freely available on the Web sites of PLoS Medicine at http://www.plosmedicine.org/, Annals of Internal Medicine at http://www.annals.org/, and Epidemiology at http://www.epidem.com/). Information on the STROBE Initiative is available at www.strobe-statement.org.

### S 6 - Pilot-test of the eCRF prior to study initiation

Table S6

| The electronic case report form (eCRF) was pilot-tested by three researchers, two ICU doctors, and the principal investigator at the coordinating site. Based on the results, the eCRF was revised before data collection began. Investigators were invited to attend an online or in-person meeting where the database was demonstrated, and general user information was distributed to all participating sites in advance. |
| --- |

| **Total score and predicted 90-day mortality risk** | | | |
| --- | --- | --- | --- |
| 0  3  4  5  6  7  8  9  10  11  12  13  14  15  16  17  18  19  20  21 | 3.3%  4.8%  5.5%  6.2%  7.1%  8.0%  9.1%  10.3%  11.6%  13.1%  14.7%  16.5%  18.4%  20.5%  22.8%  25.3%  28.0%  30.8%  33.8%  36.9% | 22  23  24  25  26  27  28  29  30  31  32  33  34  35  36  37  38  39  41  42 | 40.1%  43.4%  46.7%  50.1%  53.5%  56.9%  60.2%  63.4%  66.4%  69.4%  72.2%  74.8%  77.3%  79.6%  81.7%  83.7%  85.4%  87.0%  89.8%  91.0% |
|  |  |  |  |
|  |  |  |  |

### S 7 - Simplified Mortality Score for the Intensive Care Unit (SMS-ICU)

Table S7

|  | **Points** |
| --- | --- |
| **Age** | |
| ≤ 39 years  40 – 59 years  60 – 79 years  ≥ 80 years | 0  5  10  13 |
| **Lowest systolic blood pressure** | |
| ≤ 49 mmHg  50 – 69 mmHg  70 – 89 mmHg  ≥ 90 mmHg | 6  5  3  0 |
| **Acute surgical admission** | |
| No  Yes | 3  0 |
| **Haematological malignancy or metastatic cancer** | |
| No  Yes | 0  7 |
| **Vasopressors/inotropes^1^** | |
| No  Yes | 0  4 |
| **Respiratory support^2^** | |
| No  Yes | 0  5 |
| **Renal replacement therapy^3^** | |
| No  Yes | 0  4 |
| **Total score** | 0-42^4^ |

*Reproduced from a previous paper. (1)*

*^1^ Continuous use of any vasopressor or inotrope.*

*^2^ Use of respiratory support, including invasive or non-invasive respiratory support and continuous use of continuous positive airway pressure (CPAP). Intermittent use of CPAP is not considered respiratory support.*

*^3^Use of renal replacement therapy includes any renal replacement therapy, whether chronic or acute, including continuous renal replacement therapy and intermittent haemodialysis, including the days in between intermittent haemodialysis.*

*^4^ Points assigned for the different variables in the score. It is not possible to obtain a total score of 1, 2 or 40 points. The worst value recorded during the first day in the ICU is used. (1, 2)*

###

### S 8 - Baseline variables and definitions

Table S8a

**Unit evaluation/site variables**

| Site characteristics |  |
| --- | --- |
| Type of hospital   - General^1^ - Specialised^2^ |  |
| Type of ICU   - Medical - Surgical - Mixed   Number of ICU beds open for admission |  |

*^1^ General: Hospital or medical centre providing licensed physicians in paediatrics, obstetrics, gynaecology, general surgery and other supporting medical services.*

*^2^ Specialised: Hospital offering similar services as described for the general centres/hospitals with additional subspeciality care such as neurosurgery, cardiac surgery, plastic surgery and transplantation.*

Table S8b

| Baseline patient and admission characteristics |
| --- |
|  |
| **General patient data**   - **Sex (male/female)** - **Age (years)** - **Hospital admission date (dd.mm.yyyy)** - **ICU admission date and time (dd.mm.yyyy and hh:mm)** - **Source of admission**   - Emergency department or prehospital setting (Any Accident/Emergency /Casualty/Acute department in same or another hospital or direct admission to the ICU by an ambulance service or similar)   - General ward (Any location in the same or other hospital not covered in the other 3 categories)   - Operating or recovery room (Any surgical theatre, endoscopy and angiography suite and any recovery facilities observing patients following invasive procedures)   - Another ICU - **Main reason for ICU admission** - Neurological condition - Respiratory failure - Circulatory failure - Renal failure - Liver failure - GI bleeding - Other - **Elective surgery**   *Surgery during current hospitalisation scheduled 24 hours or more before surgery*   - **Emergency surgery**   *Surgery during current hospitalisation scheduled 24 hours or less (including surgery performed at other hospitals) before surgery*   - **Abdominal surgery**   Any abdominal surgery (*Any laparotomy or laparoscopic surgery involving the organs or structures within the abdominal cavity including structures in the pelvic region*) during current hospital admission prior to ICU admission. (Endoscopic procedures are NOT considered surgery in this setting).   - **History of bariatric surgery**   *Includes gastric bypass, gastric sleeve, gastric banding, gastric balloon*  **Comorbidities with definitions**   1. **Chronic pulmonary disease**   *Any history of chronic obstructive pulmonary disease, asthma, or other chronic lung disease or if the patient was treated at time of hospital admission with any relevant drug indicating chronic pulmonary disease, e.g. albuterol, levalbuterol, salmeterol, formoterol, arformoterol, indacaterol, vilanterol, olodaterol, tiotropium, aclidinium, umeclidinium, glycopyrronium, budesonide and fluticasone.*   1. **History of severe heart failure (NYHA 3-4) or myocardial infarction**   *History of previous myocardial infarction, invasive intervention for coronary artery disease, stable or unstable angina, NYHA class 3 or 4 or measured LVEF < 40%.*  *NYHA class 3: marked limitation of physical activity. Comfortable at rest. Less than ordinary activity causes fatigue, palpitation, shortness of breath or chest pain.*  *NYHA class 4: symptoms of heart failure at rest. Any physical activity causes further discomfort.*   1. **History of chronic liver failure**   *The presence of one or more of the following:*   - *portal hypertension* - *cirrhosis proved by biopsy, CT scan or ultrasound* - *history of variceal bleeding* - *hepatic encephalopathy in the past medical history* - Ascites (non-malignant) in past medical history  1. **History of chronic renal failure**   *Need for chronic renal support or S-creatinine > 3.6 g/dL / 300 μmol/L prior to hospital admission.*   1. **Diabetes**   *Treatment at the time of hospital admission with any relevant drug indicating diabetes, e.g., insulin, alpha-glucosidase inhibitors, biguanides (metformin), dipeptidyl peptidase-4 inhibitors (e.g., Januvia), Glucagon-like peptide-1 receptor agonists, meglitinides, sodium-glucose transporter 2 inhibitors, sulfonylureas, thiazolidinediones.*  **Treatments before ICU admission**   - Treatment with a prokinetic agent   *Treatment with a prokinetic agent (erythromycin, metoclopramide, domperidone or prucolapride)*   - Treatment with parenteral nutrition   *Treatment with parenteral nutrition on the ICU Day of inclusion (within 24 hours of ICU admission).*  **Simplified Mortality Score for the Intensive Care Unit (SMS-ICU) (not included in the baseline variables)^1^**   1. **Lowest systolic blood pressure (mmHg)**   *Worst value registered in the 24 h prior to inclusion*   1. **Hematologic malignancy or metastatic cancer**   *History of malignant hematologic disorder or metastatic cancer in medical records.*   1. **Vasopressor/inotropes**   *Continuous use of vasopressors/inotropes (i.e., not boluses) on the ICU day of inclusion*  *(E.g., epinephrine, norepinephrine, dobutamine, dopamine, phenylephrine, metaraminol, milrinone, levosimendan, angiotensin II, and vasopressin or one of its analogues)*   1. **Respiratory support**   *Use of respiratory support, including invasive or non-invasive respiratory support and continuous use of continuous positive airway pressure (CPAP) on the ICU day of inclusion.*   1. **Renal replacement therapy**   *Use of any renal replacement therapy, whether chronic or acute, including continuous renal replacement therapy and intermittent haemodialysis, including up to three days between intermittent haemodialysis on the ICU day of inclusion.* |
|  |
|  |
|  |

^1^ *Variables for the SMS-ICU score not otherwise included in the baseline variables*

*ICU: Intensive Care Unit, GI: gastrointestinal, NYHA: New York Heart association, LVEF: left ventricular ejection fraction, CT: computed tomography scan*

### S 9 - Daily variables and definitions

Table S9

| **Date** |
| --- |
| - **Date of the dayform (dd-mm-yyyy)** |
| **Life support** |
| - **Continuous treatment with a vasopressor or inotropic agents**   *The use of intermittent boluses was not considered a continuous infusion.* *(E.g., epinephrine, norepinephrine, dobutamine, dopamine, phenylephrine, metaraminol, milrinone, levosimendan, angiotensin II, and vasopressin or one of its analogues)*   - **Treatment with continuous/intermittent renal replacement therapy**   *Any renal replacement therapy, whether chronic or acute, including continuous renal replacement therapy and intermittent haemodialysis, including up to three days in between intermittent haemodialysis*   - **Invasive mechanically ventilated**   *Invasive mechanical ventilation is defined as the use of positive pressure ventilation using a ventilator via a cuffed tube (oral, nasal or tracheostomy). CPAP is NOT invasive mechanical ventilation* |
| **Potential Serious adverse events** |
| - **Cardiac arrest** - **Cardiac arrhythmias requiring pharmacological treatment**   *Any cardiac arrhythmia requiring pharmacological treatment, including supraventricular tachycardia, ventricular tachycardia, ventricular fibrillation, or bradycardia*   - **Extrapyramidal symptoms requiring pharmacological treatment**   *Akathisia, dystonia, parkinsonism, or tardive dyskinesia* *requiring pharmacological treatment*   - **Severe diarrhoea requiring treatment**   *A volume of ≥ 1000 ml/day and/or if severe diarrhoea is noted in the medical record and requires treatment, e.g., fluids, stopping/pausing medication, or pharmacological therapy.*   - **Vomiting with clinically significant aspiration requiring treatment**   *Entry of solid material into the trachea and lungs, resulting in clinical deterioration with need for treatment (pharmacological or non-pharmacological) and/or aspiration requiring treatment, was noted in the medical record.* |
| **Nutrition** |
| - **Any enteral and/or oral intake** |
| **Treatment with prokinetic agents** |
| - **Type of prokinetic agents**   **Metoclopramide, erythromycin, domperidone or prucolapride**   - **Dose (mg)** - **Times pr day** - **Intravenous, orally or both** |

*Only variables used in the present study are listed above. For full details on all daily variables collected in the PATIENCE cohort, please refer to the published protocol. (3)*

*CPAP: Continuous Positive Airway Pressure*

### S 10 - Follow-up variables and definitions

Table S10

| **Vital status** |
| --- |
| - **Death within 90 days after inclusion**   Death from any cause  **If yes**   - - Date of death (dd-mm-yyyy) |
| **Hospital discharge(s)/ readmission(s)** |
| - **Discharged alive from the hospital within 90 days after inclusion**   *Discharge from index hospitalisation (the hospitalisation during wich the patient was included in the PATIENCE study)*  If yes   - - **Date of index hospital discharge (dd-mm-yyyy)** - **Additional hospitalisations within 90 days after inclusion**   Any non-psychiatric hospitalisations after discharge from the index hospitalisation within the 90-day follow-up  If yes   - - Dates of additional hospital admissions and discharges (dd-mm-yyyy) |

### S 11 - Outcome measures

Table S11

| Primary outcome |
| --- |
| Proportion of patients who receive prokinetic agents  *Proportion of patients who receive any of the following prokinetic agents: erythromycin, metoclopramide, domperidone, or prucalopride during any intensive care unit (ICU) stay within the data collection period.* |
| Secondary Outcomes |
| 90-day mortality *Death from any cause within 90 days of ICU admission.* |
| Days alive without life support  *The total number of days alive without the use of invasive mechanical ventilation, continuous infusion of vasopressors or inotropic agents and renal replacement therapy (including up to three days in between intermittent haemodialysis). The total number of days alive without the use of life-support was calculated as the total number of days with the use of life-support subtracted from the total number of days alive during the study period of 90 days.* |
| Days alive and out of ICU  *The total number of days alive and out of ICU was calculated as the total number of days spent in the ICU subtracted from the total number of days alive during the 90-day study period.* |
| Days alive and out of hospital  *The total number alive and out of hospital was calculated as the total number of days spent in the hospital subtracted from the total number of days alive during the 90-day study period.* |
| Specific serious adverse events (SAEs) during ICU stay  *Total number of patients with one or more SAEs during ICU stay. These events represent potential adverse reactions to prokinetic agents. They were recorded for all patients, regardless of whether they received prokinetic treatment.*  SAEs:   - Cardiac arrest - Cardiac arrhythmias requiring pharmacological treatment - Extrapyramidal symptoms requiring pharmacological treatment - Vomiting with clinically significant aspiration requiring treatment |

### S 12 - Deviations from the protocol

Table S12

| **Additions/changes** | **Reason** |
| --- | --- |
| Change of primary admission groups | Admission groups were categorised as respiratory, neurological, circulatory, or other (including liver failure, renal failure, gastrointestinal bleeding, and other), based on available data and patient distribution. This differs from the protocol, which prespecified grouping by admission type (acute abdominal surgery, other acute surgery, or medical). However, these categories were not collected as admission reasons during data entry and could therefore not be applied. |
| Inclusion of abdominal surgery and acute surgery as separate variables in the analyses | In the protocol, the prespecified admission reasons were acute abdominal surgery and acute surgery other than abdominal. In the available dataset, these were represented by the variables abdominal surgery and acute surgery, which were used as separate variables in the final analyses. |

### S 13 – Best and worst case scenarios

Table S13

| **Secondary analysis scenarios**  Three scenarios were applied to the secondary outcome analyses:   1. **Worst-case scenario (transfers)** – Patients transferred to non-participating ICUs were assumed to require ICU care and life support for all days from transfer until hospital discharge. 2. **Worst-case (deaths and transfers)** - Deaths were assigned zero days, and transfers were assumed to require ICU care and life support until hospital discharge 3. **Best-case scenario** – only observed days with available data were included, with no assumptions made after transfer, effectively treating patients as not having received ICU care or life support after transfer.   **Model adjustments**  All models were adjusted for country, SMS-ICU score, number of comorbidities (0,1,2 or ≥3), abdominal surgery, acute surgery, and ICU admission type.  SMS-ICU was modelled as a continuous variable in line with the pre-specified analysis plan. Based on model diagnostics, a log transformation of SMS-ICU was applied in the analyses of days alive out of ICU and days alive without life support analyses to address non-linearity. This was not necessary in the other analyses. (3) |
| --- |

### S 14 - Model diagnostics and assumption checks

Table S14

| Proportional hazards assumptions were evaluated using scaled Schoenfeld residuals, and linearity of SMS-ICU was assessed by including both linear and log(x+1)-transformed terms in the models; the log-transformed terms were kept if they were statistically significant.  **Time to treatment models -** No violations of the proportional hazard assumptions were identified.  **90-day mortality model** – Initial violations were observed for SMS-ICU and country. After adding logarithmic time-transformations for these, further violations were identified for the number of comorbidities and admission type, which were addressed in the same way. A minor residual violation remained for the respiratory admission category (p=0.035); this was accepted for this adjustment variable.  **Serious adverse events model** - Violations for SMS-ICU, number of comorbidities, and ICU admission type were addressed with logarithmic time-transformations, with a minor residual violation for the respiratory admission category (p=0.035); this was accepted for this adjustment variable.  No evidence of non-linearity for SMS-ICU in any of the above models |
| --- |

### S 15 - Missing data

Table S15a

**Missing baseline variables**

| Variable | Missing n (%) |
| --- | --- |
| Blood pressure | 2 (0.14%) |
| Chronic liver failure | 1 (0.07%) |
| Chronic pulmonary disease | 1 (0.07%) |
| Ischemic heart disease or heart failure | 1 (0.07%) |
| Parenteral nutrition at ICU admission | 1 (0.07%) |
| Elective surgery | 1 (0.07%) |

*Seven patients had one missing baseline variable (0.5%). For those with missing blood pressure, no measurement was recorded within the first 24 hours of ICU admission. Only variables with missing values are included in the table*.

ICU: Intensive Care Unit

Table S15b

**Missing daily variables**

| Daily variable | Number of patients | Number of ICU days with missing data |
| --- | --- | --- |
| Infusion of vasopressor | 1 | 1 |
| Invasive mechanical ventilation | 1 | 1 |
| Renal replacement therapy | 1 | 1 |
| Cardiac arrhythmias | 1 | 1 |
| Extrapyramidal symptoms | 1 | 1 |

*Three patients (0.2%) had one or more daily variables missing. In one patient, all life support variables were missing for a single intensive care unit day. Only variables with missing values are shown*.

ICU: Intensive Care Unit

Table S15c

| **Patients transferred to non-participating intensive care units (ICUs)**  A total of 66 (4.6%) were transferred directly from a participating ICU to a non-participating ICU during the 90-day follow-up period.  **Handling:**   1. Regression analyses: a best-case approach was applied, including only observed days and making no assumptions about the period after transfer, effectively treating patients as not receiving ICU care or life support after transfer. Secondary analyses were made with a worst-case approach, assigning ICU care and life support use for all days from the time of transfer to hospital discharge for patients transferred to non-participating ICUs. 2. Cox models: follow-up was censored at the time of transfer. 3. Descriptive analyses: no events were assumed to occur during time spent in the non-participating ICUs.   **Lost to follow up**  One patient (0.1%) was lost to follow-up, and information on additional admissions was unavailable for three patients (0.3%).  **Handling**: In accordance with the protocol, patients lost to follow-up were censored in the Cox models and excluded from regression analyses. |
| --- |

### S 16 - Study flow diagram


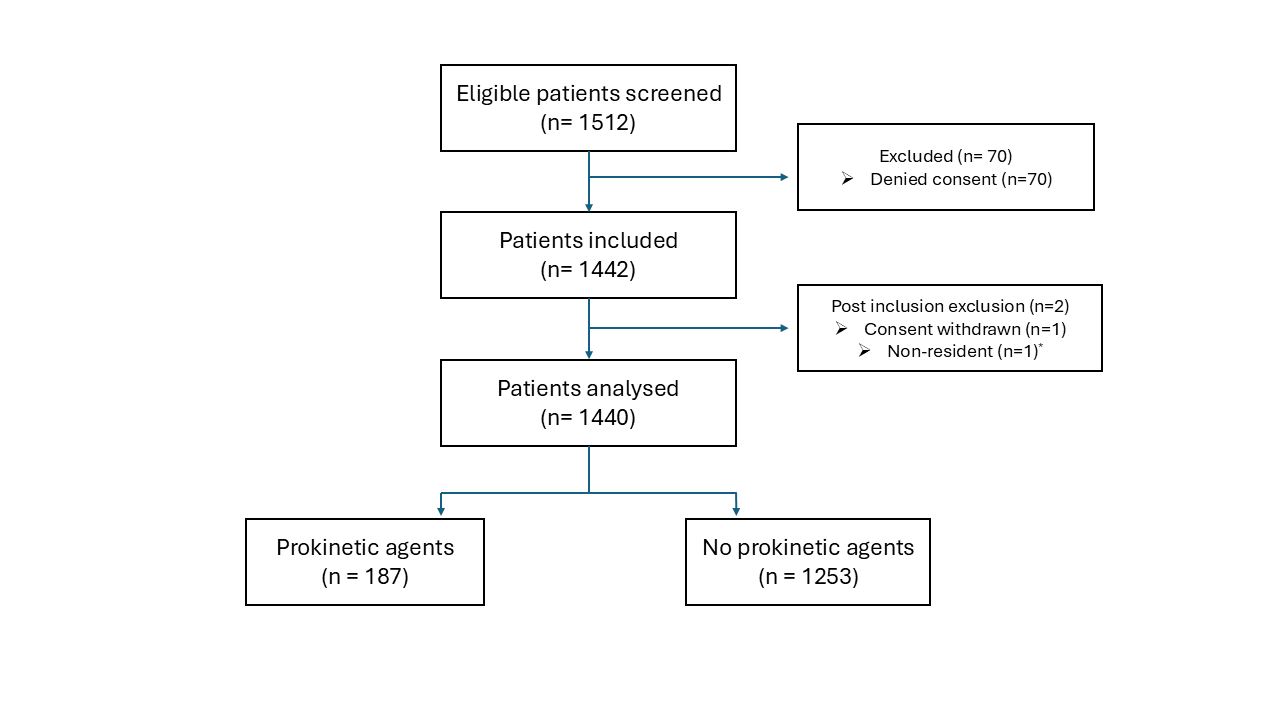


**Figure S1:** *Only patients meeting all inclusion criteria were screened for inclusion. Patients already admitted to the intensive care unit at the start of the inception periods were not screened. One patient withdrew consent after inclusion and was deleted from the database before analysis.*

**The patient was included in Denmark, but was not a resident and was excluded before analysis due to a lack of approval to use the data*

### S 17 - Site characteristics

Table S17

|  | **ICUs (n=56)^1^** |
| --- | --- |
| **Type of hospital**  *General^2^*  *Specialised^3^* | 30 (54%)  26 (46 %) |
| **Type of ICU**  *Medical*  *Surgical*  *Mixed* | 2 (4 %)  4 (7%)  50 (89%) |
| **Number of beds open for admission**  *< 10*  *10-19*  *20-29*  *≥ 30*  **Median (IQR; range)** | 16 (29%)  20 (36%)  11 (20%)  9 (16%)  12 (8-24; 5-81) |

*^1^There were no missing data*

*^2^General: Hospital or medical centre providing licensed physicians in paediatrics, obstetrics, gynaecology, general surgery and other supporting medical services.*

*^3^ Specialised: Hospital offering similar services as described for the general centres/hospitals with additional subspeciality care such as neurosurgery, cardiac surgery, plastic surgery and transplantation.*

*ICU: Intensive Care Unit ; IQR: Interquartile Range*

### S 18 - Length of ICU stay

**
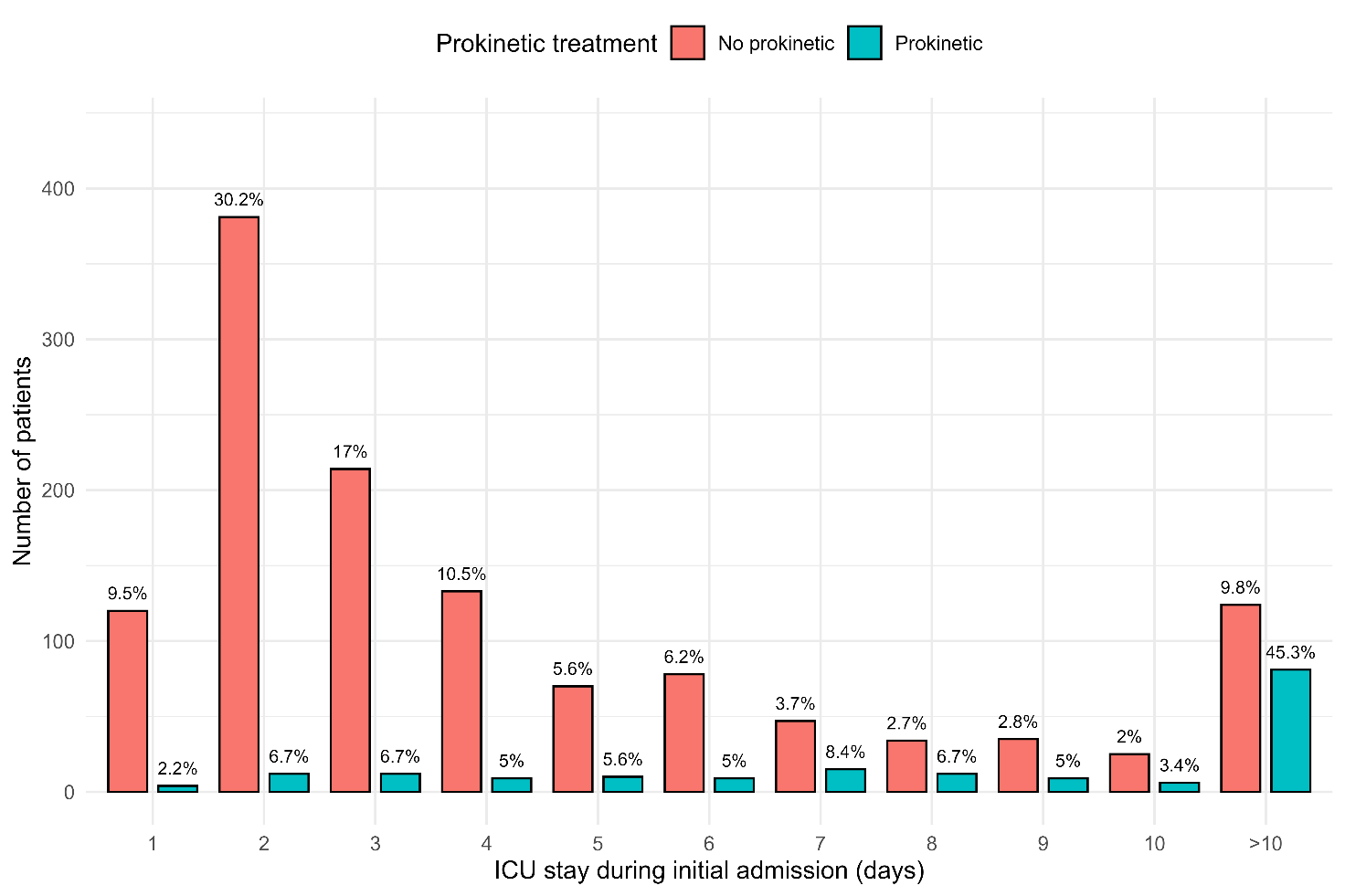
Fig S2 length of index ICU-stay**

***Figure S2*** *N=1440. Length of ICU stay during first (index) ICU admission stratified by prokinetic treatment. Sixty-six patients (4.6%) were transferred to a non-participating ICU, and ICU stay was assumed to have ended at the time of transfer. Percentages are calculated within each treatment group.*

### S 19 - Data on prokinetics

**Dose and frequency for the use of metoclopramide**


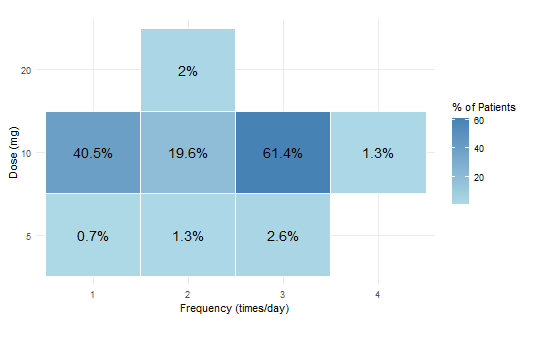


***Figure S3*** *Metoclopramide dose-frequency combinations among treated patients (n = 153, 81.8%). Patients who received metoclopramide either as monotherapy or in combination with other prokinetic agents are included. Each patient may contribute to multiple dose-frequency combinations if different doses or frequencies were administered during their intensive care unit stay. Eight patients received metoclopramide orally.*


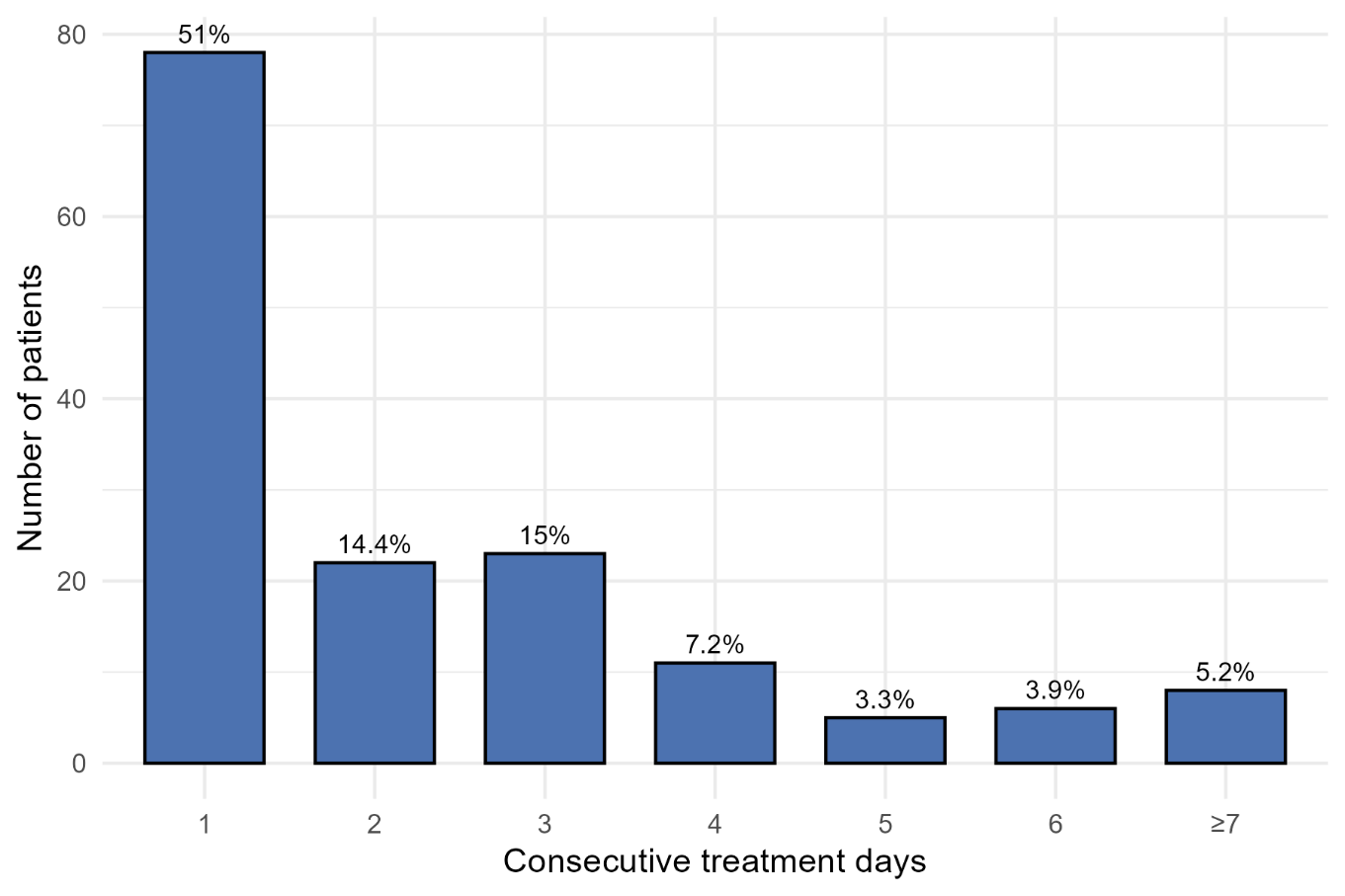
**Duration of treatment for metoclopramide**

**Figure S4** *Consecutive days of the first metoclopramide treatment episode (n=153, 81.8%). Includes all patients who received metoclopramide either as monotherapy or in combination with other prokinetic agents.*

**Dose and frequency for the use of erythromycin**


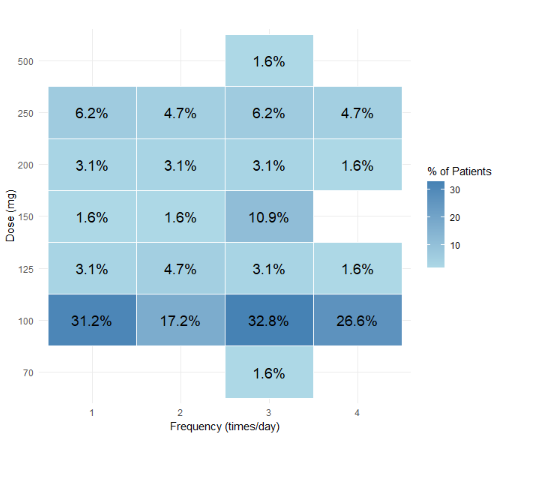


***Figure S5*** *Erythromycin dose-frequency combinations among treated patients (n = 64, 34.2%). Includes patients who received erythromycin either as monotherapy or in combination with other prokinetic agents. Each patient may contribute to multiple dose-frequency combinations if different doses or frequencies were administered during intensive care unit stay. A dose of 70 mg was administered to one patient with the indication for obstipation. All but one patient received erythromycin intravenously.*

**Duration of treatment for erythromycin**


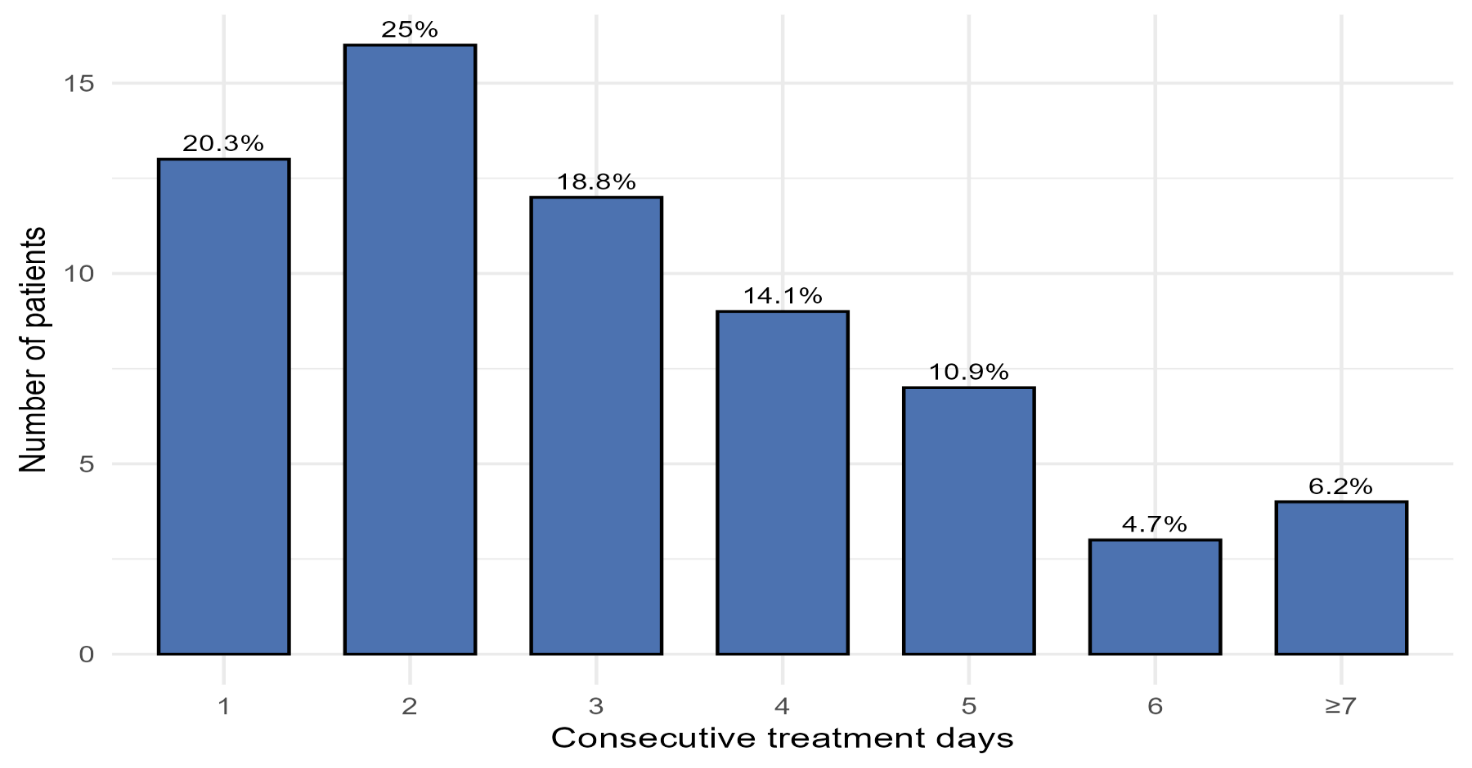


***Figure S6*** *N= 64, 34.2%. Consecutive days of the first erythromycin treatment episode. Includes all patients who received erythromycin either as monotherapy or in combination with other prokinetic agents.*

### S 20 - Time to first prokinetic treatment

**Time to first prokinetic treatment (index admission)**


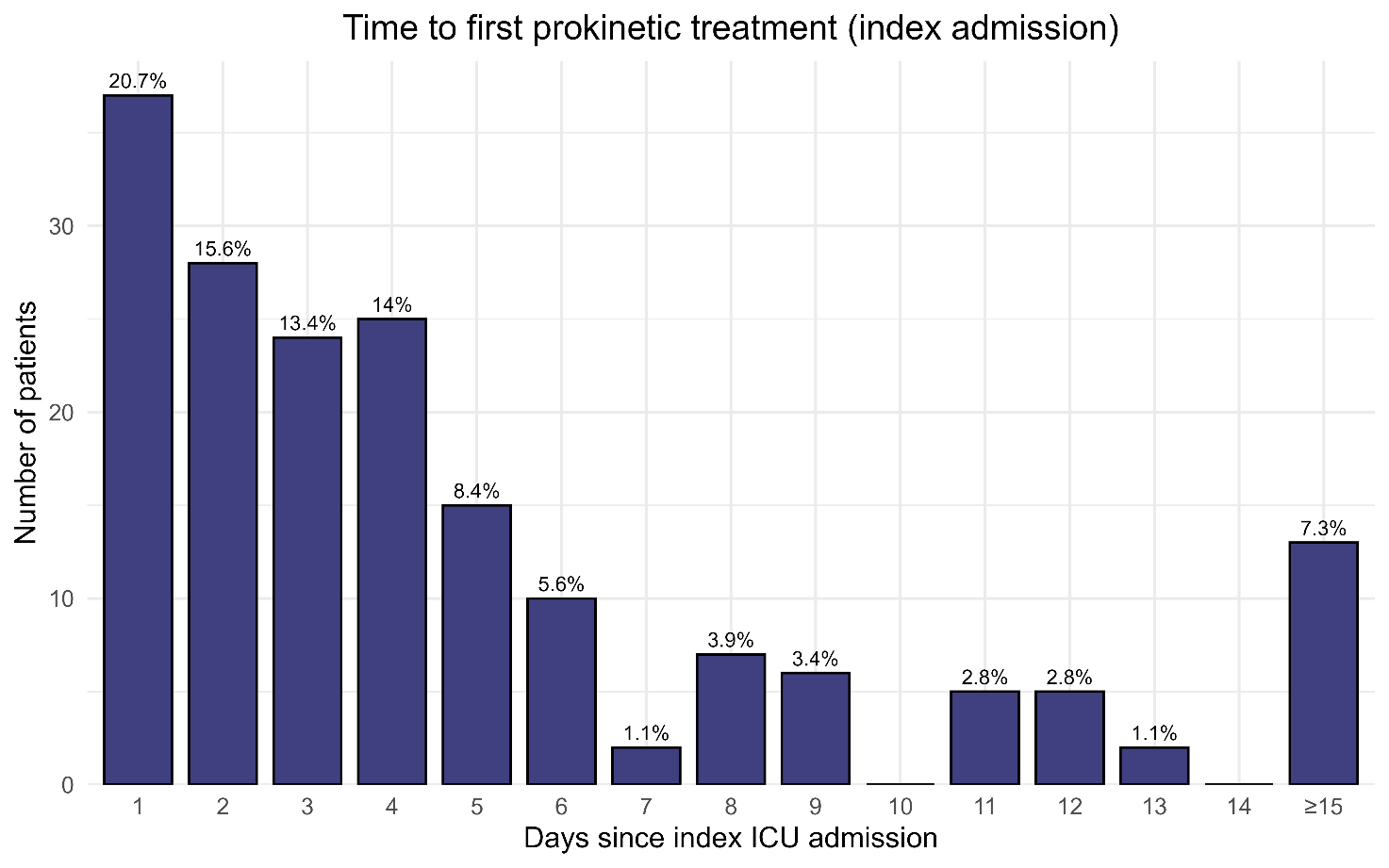


***Figure S7*** *Time to first prokinetic treatment during index ICU admission (n=179, 95.7%). Bars show the distribution of days from index admission to first treatment. Eight patients received prokinetic agents only during readmission (treatment started on days 1-13 after readmission) and are not included in the figure.*

*ICU: Intensive Care Unit*

### S 21 - Prokinetic use by country

**Prokinetic patterns by country**


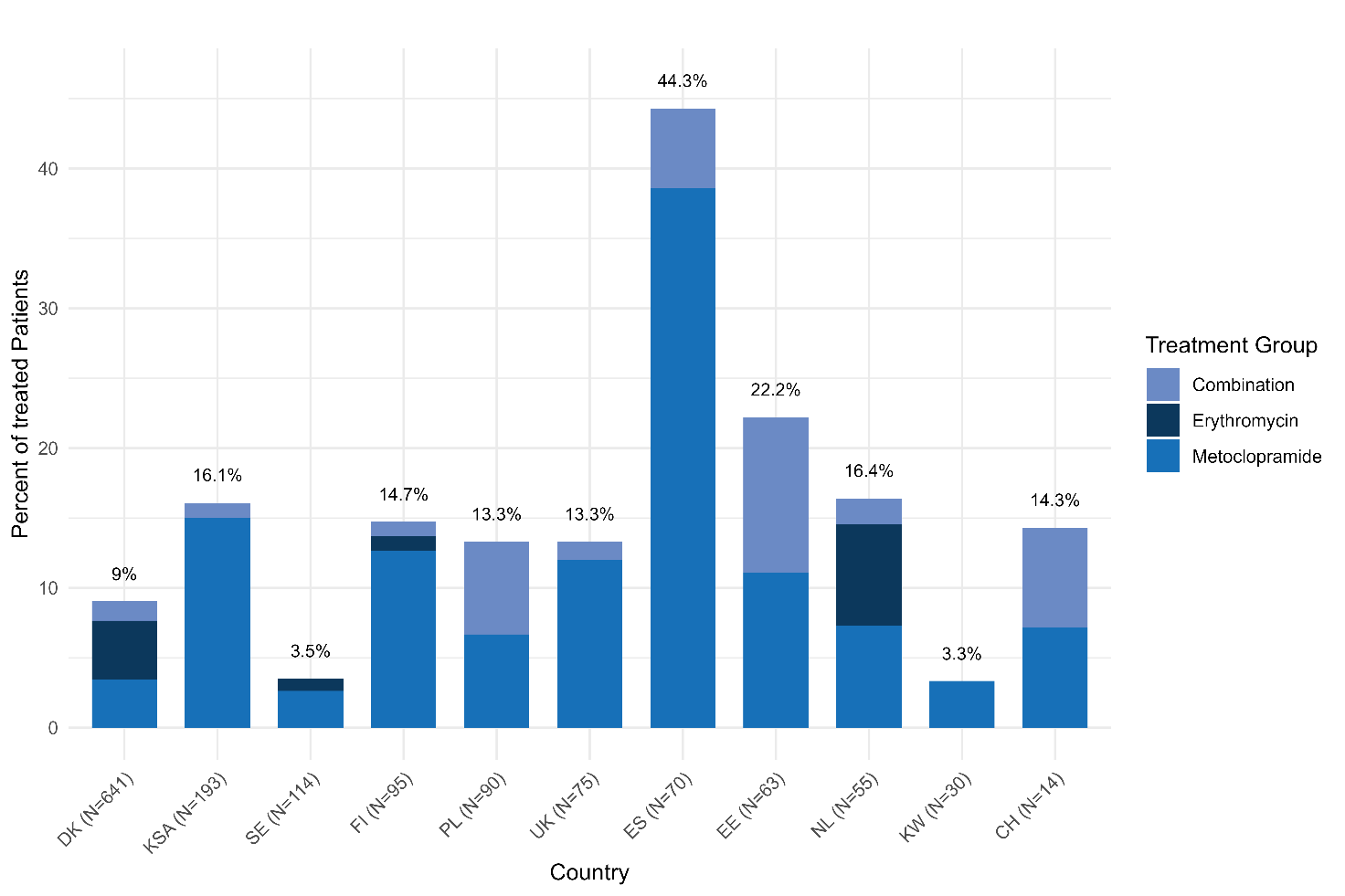


**Figure S8** *Prokinetic treatment patterns by country. Percentages represent the proportion of all included patients from each country who received metoclopramide, erythromycin, or combination therapy. Two patients (one from UK and one from KSA) received domperidone. In the combination group, five patients (two from DK, one from NL, one from EE, and one from KSA) received the agents on different days*

*DK; Denmark, KSA; Kingdom of Saudi Arabia, SE; Sweden, FI; Finland, PL; Poland, UK; United Kingdom, ES; Spain, EE; Estonia, NL; Netherlands, KW; Kuwait, CH; Switzerland*

### S 22 - Use of life support

Table S22

**Use of life support during ICU stay**

|  | All patients (n=1,440) | No prokinetic agents (n=1,253) | Prokinetic agents  (n=187) |
| --- | --- | --- | --- |
| Invasive mechanical ventilation | 814 (56.5%) | 689 (55.0%) | 125 (66.8%) |
| Vasopressors | 758 (52.6%) | 646 (51.6%) | 112 (59.9%) |
| Renal replacement therapy | 100 (7%) | 84 (6.7%) | 16 (8.6%) |

*Values are the number of patients (%). Patients may be counted in more than one category. Percentages are calculated within each treatment group.*

### S 23 - Descriptive overview and regression analyses of secondary outcomes

Table S23a

**Descriptive overview of secondary outcomes – best case (observed days)**

| *Outcome* | *Overall*  *(n= 1,436)*  *Median (IQR)* | *No prokinetic agents*  *(n=1,250)*  *Median (IQR)* | *Prokinetic agents*  *(n=186)*  *Median (IQR)* |
| --- | --- | --- | --- |
| *Days alive without life support* | *87 (57-89)* | *88 (61-90)* | *80 (34-86)* |
| *Days alive out of ICU* | *84 (49-88)* | *86 (59-88)* | *73 (30-82)* |
| *Days alive out of hospital* | *65 (0-81)* | *69 (0-81)* | *35 (0-65)* |

*Secondary outcomes stratified by prokinetic use. Values are medians with interquartile range (IQR) within the 90-day follow-up period. Four patients were lost to follow-up.*

*ICU: Intensive Care Unit; IQR: Interquartile Range*

Table S23b

**Descriptive overview secondary outcomes - worst case (observed days)**

| *Outcome* | *Overall*  *(n= 1,436)*  *Median (IQR)* | *No prokinetic agents (n=1,250)*  *Median (IQR)* | *Prokinetic agents (n=186)*  *Median (IQR)* |
| --- | --- | --- | --- |
| *Days alive without life support* | *74 (7-84)* | *77 (8-85)* | *50 (6-76)* |
| *Days alive out of ICU* | *71(1-82)* | *74 (1-82)* | *43 (0-70)* |
| *Days alive out of hospital* | *65 (0-81)* | *69 (0-81)* | *35 (0-65)* |

*Secondary outcomes stratified by prokinetic use. Values are medians with interquartile range (IQR) within the 90-day follow-up period. Four patients were lost to follow-up.*

*ICU: Intensive Care Unit; IQR: Interquartile Range*

Table S23c

***Descriptive overview secondary outcomes - worst case (dead=0)***

| *Outcome* | *Overall*  *(n= 1,436)*  *Median (IQR)* | *No prokinetic agents (n=1,250)*  *Median (IQR)* | *Prokinetic agents (n=186)*  *Median (IQR)* |
| --- | --- | --- | --- |
| *Days alive without life support* | *87 (57-89)* | *88 (61-90)* | *80 (34-86)* |
| *Days alive out of ICU* | *84 (49-88)* | *86 (59-88)* | *73 (30-82)* |
| *Days alive out of hospital* | *65 (0-81)* | *69 (0-81)* | *35 (0-65)* |

*Secondary outcomes stratified by prokinetic use. Values are medians with interquartile range (IQR) within the 90-day follow-up period. Four patients were lost to follow-up.*

*ICU: Intensive Care Unit; IQR: Interquartile Range*

Table S23d

**Secondary regression analyses of days alive outcomes**

| Outcome | Best case (observed days)  *Adjusted MD (95% CI), p-value* | Worst case (dead = 0 days)  *Adjusted MD (95% CI), p-value* | Worst case (observed days)  *Adjusted MD (95% CI), p value* |
| --- | --- | --- | --- |
| *Days alive without life support* | -5.59 (-11.05 to -0.28),  p= 0.042 | -4.88 (-10.82 to 1.01),  p= 0.107 | -5.93 (-8.00 to -4.00),  p < 0.001 |
| *Days alive out of ICU* | -8.08 (-13.53 to -2.92),  p = 0.003 | -6.97 (-12.67 to -1.36),  p= 0.016 | -8.41 (-10.60 to -6.34),  p < 0.001 |
| *Days alive out of hospital* | -12.98 (-18.12 to -7.90),  p < 0.001 | -13.08 (-18.24 to -7.86),  p < 0.001 | -13.94 (-18.72 to -9.23),  p < 0.001 |

*Secondary outcomes in scenarios described above.*

*Adjusted MD accounting for SMS-ICU, number of comorbidities, admission reason, acute abdominal surgery, acute surgery other than abdominal, and country. Estimates are derived from linear models using non-parametric bootstrapping with 50,000 resamples.
ICU: Intensive Care Unit; IQR: Interquartile Range; MD: mean differences, CI: Confidence interval*

### References

1. Granholm A, Perner A, Krag M, et al.: Development and internal validation of the Simplified Mortality Score for the Intensive Care Unit (SMS-ICU) [Internet]. *Acta Anaesthesiologica Scandinavica* 2018

2. Granholm A, Perner A, Krag M, et al.: External validation of the Simplified Mortality Score for the Intensive Care Unit (SMS-ICU). *Acta Anaesthesiologica Scandinavica* 2019; 63:1216–1224

3. Crone V, Møller MH, Perner A, et al.: Prokinetic agents in adult intensive care unit patients ( PATIENCE )—An international inception cohort study protocol. *Acta Anaesthesiol Scand* 2024; 68:1601–1606
